# Allele-specific binding of ZFP57 in the epigenetic regulation of imprinted and non-imprinted monoallelic expression

**DOI:** 10.1186/s13059-015-0672-7

**Published:** 2015-05-30

**Authors:** Ruslan Strogantsev, Felix Krueger, Kazuki Yamazawa, Hui Shi, Poppy Gould, Megan Goldman-Roberts, Kirsten McEwen, Bowen Sun, Roger Pedersen, Anne C. Ferguson-Smith

**Affiliations:** Department of Physiology Development and Neuroscience, University of Cambridge, Downing Site, Cambridge, CB2 3EG UK; Bioinformatics Department, Babraham Institute, Cambridge, CB22 3AT UK; The Anne McLaren Laboratory for Regenerative Medicine, University of Cambridge, Cambridge, CB2 0SZ UK; Present address: Department of Genetics, University of Cambridge, Downing Street, Cambridge, CB2 3EH UK; Present address: Epigenetics ISP, Babraham Institute, Cambridge, CB22 3AT UK

## Abstract

**Background:**

Selective maintenance of genomic epigenetic imprints during pre-implantation development is required for parental origin-specific expression of imprinted genes. The Kruppel-like zinc finger protein ZFP57 acts as a factor necessary for maintaining the DNA methylation memory at multiple imprinting control regions in early mouse embryos and embryonic stem (ES) cells. Maternal-zygotic deletion of ZFP57 in mice presents a highly penetrant phenotype with no animals surviving to birth. Additionally, several cases of human transient neonatal diabetes are associated with somatic mutations in the ZFP57 coding sequence.

**Results:**

Here, we comprehensively map sequence-specific ZFP57 binding sites in an allele-specific manner using hybrid ES cell lines from reciprocal crosses between C57BL/6J and Cast/EiJ mice, assigning allele specificity to approximately two-thirds of all binding sites. While half of these are biallelic and include endogenous retrovirus (ERV) targets, the rest show monoallelic binding based either on parental origin or on genetic background of the allele. Parental-origin allele-specific binding is methylation-dependent and maps only to imprinting control differentially methylated regions (DMRs) established in the germline. We identify a novel imprinted gene, *Fkbp6*, which has a critical function in mouse male germ cell development. Genetic background-specific sequence differences also influence ZFP57 binding, as genetic variation that disrupts the consensus binding motif and its methylation is often associated with monoallelic expression of neighboring genes.

**Conclusions:**

The work described here uncovers further roles for ZFP57-mediated regulation of genomic imprinting and identifies a novel mechanism for genetically determined monoallelic gene expression.

**Electronic supplementary material:**

The online version of this article (doi:10.1186/s13059-015-0672-7) contains supplementary material, which is available to authorized users.

## Background

Genomic imprinting is a mechanism of gene regulation occurring in eutherian mammals and to a lesser extent marsupials [1–3]. Unlike most autosomal genes that are expressed from both parental copies, imprinted genes are transcribed from either the maternal or paternal chromosome. There are currently over 150 genes known to be imprinted in mouse, many of which are also imprinted in human [4, 5]. Correct dosage of imprinted genes is important for normal development, exemplified in mouse models [6] and by human genetic syndromes resulting from epimutation and uniparental disomy affecting imprinted loci [7–11]. Imprinted genes are mostly, though not exclusively, organized in clusters up to 4 Mb in size. Each cluster is associated with a germline differentially methylated region (germline DMR), which in all cases functions as an imprinting control region (ICR) for multiple genes in the locus. Deletion of this element is consistent with loss of imprinting at the corresponding region [12–17]. Four stages of DNA methylation occur at germline DMRs in mouse: erasure in primordial germ cells, differential establishment during male and female gametogenesis, specific targeted maintenance during pre-implantation development at a time when the majority of the genome becomes hypomethylated, and post-implantation maintenance in somatic cells, which provides the basis for heritable imprinted gene expression [3, 18].

Previous studies demonstrated a critical role for the KRAB zinc finger protein ZFP57 in the maintenance of DNA methylation at multiple imprinted germline DMRs in mice and human [19, 20]. Failure to maintain DNA methylation imprints upon maternal-zygotic deletion of the protein resulted in embryonic lethality by E16.5. Two recently published studies analyzing ZFP57 null embryonic stem (ES) cells have also shown hypomethylation at multiple imprinted loci akin to those observed in the maternal-zygotic mutants [21, 22]. Interestingly, Zuo and colleagues [22] found that re-introduction of exogenous ZFP57 in knock-out ES cells did not result in re-establishment of DNA methylation, indicating irreversible loss of epigenetic memory at these DMRs. This is consistent with studies demonstrating that methylation of a CpG within the binding motif is necessary for ZFP57 binding in vitro [21].

The mechanism by which ZFP57 mediates its function is associated with its interacting partner KAP1, also known as TRIM28 or TIF1β. A complex containing ZFP57 and KAP1 appears to co-immunoprecipitate with all three catalytically active DNA methyl transferases as well as the hemi-methylated DNA binding protein NP95/UHRF1. Moreover, wild-type but not KRAB domain-deleted ZFP57 (incapable of KAP1 interaction) could substitute for the endogenous ZFP57 in ES cell DNA methylation maintenance [22]. Finally, a maternal effect mutation of KAP1 was recently found to result in epigenetic instability, including frequent loss of methylation at the H19 ICR [23], suggesting a function for KAP1 in maintaining epigenetic marks during fertilized oocyte to embryo transition.

Utilizing an ES cell line expressing exogenous HA-tagged ZFP57, Quenneville and colleagues [21] mapped genomic binding sites of ZFP57 in a non-allele-specific manner and analyzed specific ICRs in a single hybrid ES cell line, demonstrating ZFP57 binding to the methylated allele in each case. Here we perform genome-wide allele-specific mapping of ZFP57 in mouse reciprocal hybrid ES cells using an antibody specific to the endogenous protein. Crucially, the use of reciprocal hybrid lines allowed us to distinguish true parental-origin allele-specific binding from binding determined solely by the genetic sequence. We show that allele-specific binding is exclusively at imprinted germline DMRs and not at DMRs derived somatically or germline DMRs that are not imprinting control regions. Furthermore, analysis of all ZFP57-bound germline DMRs revealed a highly predictive power to identify new imprinted loci, including a previously unreported *Fkbp6* imprinted gene on chromosome 5.

In addition, we discover that mouse strain-specific binding can be specified by sequence variation in the ZFP57 binding motif itself, and indirectly via genetically determined differential methylation. Analysis of allele-specific expression of genes associated with such non-imprinted ZFP57 binding provides the first mechanistic insight into how genetic variation might lead to monoallelic gene expression via epigenetic mechanisms regulated by ZFP57.

## Results

### Allele-specific mapping of ZFP57 binding sites

**Fig. 1:**
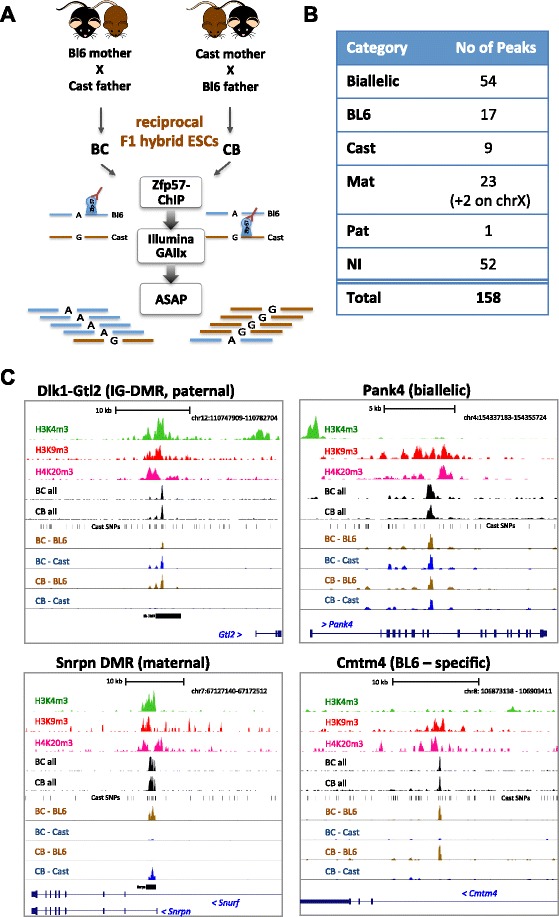
Allele-specific mapping of Zfp57 binding sites*.* **a** Schematic diagram of the experimental design. Example polymorphisms are shown for the maternal allele-specific binding of ZFP57 over BL6 (A)–Cast (G) SNP. BL6 allele (*blue line*) is bound by ZFP57 in BC and Cast (*brown line*) in the CB line. *ASAP* allele-specific alignment pipeline devised to map genome-specific reads; *ESC* embryonic stem cell. **b** Total number of peaks identified as being biallelic, parental origin-specific (maternal (*Mat*) and paternal (*Pat*)) and strain-specific (BL6 and Cast) based on Fisher’s exact test analysis of allele-specific reads under each peak. *NI* refers to non-informative sites due to lack of SNP spanning reads. **c** UCSC genome browser views (mm9 assembly) of data showing parental allele-specific binding at the paternally methylated IG-DMR (*top left*), maternally methylated *Snrpn* ICR (*bottom left*), biallelic binding site within *Pank4* gene intron on chromosome 4 (*top right*) and a strain-specific monoallelic binding within the *Cmtm4* gene intron on chromosome 8 (*bottom right*). Tracks in each panel show the following (top-down): histone modification profiles for H3K4me3, H3K9me3 and H4K20me3 [28]; combined read densities for BC and CB ZFP57 ChIP; Bl6/Cast SNPs [24]; density plots for allele-specific reads in BC and CB ChIP; position of known ICRs where found; annotated UCSC genes in the region

In order to comprehensively map parental allele-specific ZFP57 binding targets, we performed chromatin immunoprecipitation (ChIP) followed by DNA sequencing (ChIP-seq) in hybrid ES cells derived from reciprocal F1 crosses between C57BL/6J (BL6, B) and Cast/EiJ (Cast, C) mouse strains (BC and CB lines, respectively) as outlined in Fig. 1a. Expression of ZFP57 in these cells was confirmed by western blotting and found to be comparable to a commonly used E14 ES cell line (Fig. S1 in Additional file 1). ChIP-seq reads were aligned in a strain-specific manner using a purpose developed allele-specific alignment pipeline (ASAP) using ~20.5 million single nucleotide polymorphisms (SNPs) between BL6 and Cast genomes obtained from Biomart and Sanger mouse genome databases [24, 25] with an overall BL6/Cast read ratio close to one (Table 1).

**Table 1:** Summary of ASAP read alignments

	BC_Zfp57	CB_Zfp57	BC_input	CB_input
Total reads	30,194,924	29,235,782	31,025,973	30,133,611
Common aligned	11,292,411	15,300,444	16,466,916	15,791,179
Bl6-aligned	1,122,729	1,919,283	1,365,545	1,302,297
Cast-aligned	1,067,237	1,875,100	1,314,585	1,292,906
Ratio BL6/Cast	1.05	1.02	1.04	1.01

A high confidence list of total and allele-specific ZFP57 binding sites was generated (Fig. S2 in Additional file 1) followed by extensive independent experimental validation (Fig. S3 in Additional file 1). In total, 158 endogenous ZFP57 targets were identified in ES cells (Additional file 2). Strain-specific alignments determined the allele specificity of each peak and we statistically assessed read ratios (*p* < 0.0001) either towards the parental or strain-specific origin of the allele. The remainder of the peaks were classed as being either biallelic or non-informative. In total, significant allelic assignments were made for approximately two-thirds of binding sites (Additional file 2; Fig. 1b). A larger proportion of binding events (54) occurred on both parental alleles, and we identified an equal number of parental-origin (25 maternal/1 paternal) and strain-specific (17 BL6/9 Cast) instances of monoallelic binding. We found ZFP57 bound all informative imprinting controlling germline DMRs associating with the methylated allele (Fig. 1 and Table 2). In fact, all of the parental allele-specific sites were located within known imprinted clusters marking the germline but not somatic DMRs, except for two found on chromosome X (expected maternal allele in these two male cell lines). Examples of biallelic, parental and strain-specific binding are illustrated in Fig. 1c.

**Table 2:** Summary of ZFP57 binding within ICRs

Chr	Start	End	Gene	ChIP-seq peaks	TGCCGCR motif	TGCCGCY motif	ChIP-seq allele	SNP-Pyro allele
1	63246657	63247300	Gpr1	1	1	2	Mat	Mat
1	63305566	63315360	Zdbf2	-	-	2	-	-
2	157385609	157387535	Nnat/Peg5	1	4	2	N/A	ND
2	152512421	152513169	Mcts2	1	3	-	Mat	ND
2	174119863	174126564	Gnas_ProXL	2	7	7	Mat	Mat
2	174150877	174154638	Gnas_Ex1A	-	-	4	-	-
5	135825859	135825980	Fkbp6	1	1	-	Bi	Bi
6	4696743	4699483	Peg10	1	3	-	N/A	ND
6	30684932	30689966	Mest	3	8	4	Mat	ND
6	58856396	58857391	Nap1l5	1	4	1	N/A	ND
7	67147381	67151583	Snrpn	3	9	1	Mat	Mat
7	6679787	6684257	Peg3	2	7	1	Mat	Mat
7	135830870	135832249	Inpp5f_v2	2	6	-	Mat	ND
7	149764673	149771930	Igf2/H19	1	5	1	N/A	Pat
7	150480736	150482810	KvDMR1	1	3	-	Mat	Mat
8	125387861	125390344	Cdh15	1	2	-	Bi	ND
9	89767090	89775128	Rasgrf1	1	5	4	N/A	Pat
10	12809697	12812131	Zac1	1	6	-	Mat	Mat
12	110762703	110773093	IG-DMR	1	8	2	Pat	Pat
11	11925127	11927100	Grb10	1	3	1	Mat	Mat
11	22871610	22874212	Zrsr1	2	-	2	Mat	ND
15	72639707	72641342	Peg13	3	5	-	Mat	ND
17	12934169	12935816	Igf2r	4	7	1	Mat	ND
18	13130435	13133510	Impact	2	5	-	Mat	ND

For each locus, the number of ZFP57 ChIP-seq peaks, underlying TGCCGCR/Y binding motifs and allele-specificity are shown. Start and end points of germline DMRs are taken from [31, 32, 40]. All coordinates are NCBI/mm9 genome assembly. *Bi* biallelic Zfp57 binding at Cdh15 DMR consistent with biallelic methylation reported in ES cells [40], *Mat* maternal, *N/A* no available SNPs to distinguish allele specificity, *ND* pyrosequencing assay not conducted for this locus, *Pat* paternal

### ZFP57 targets are associated with repressive histone marks and DNA methylation

**Fig. 2:**
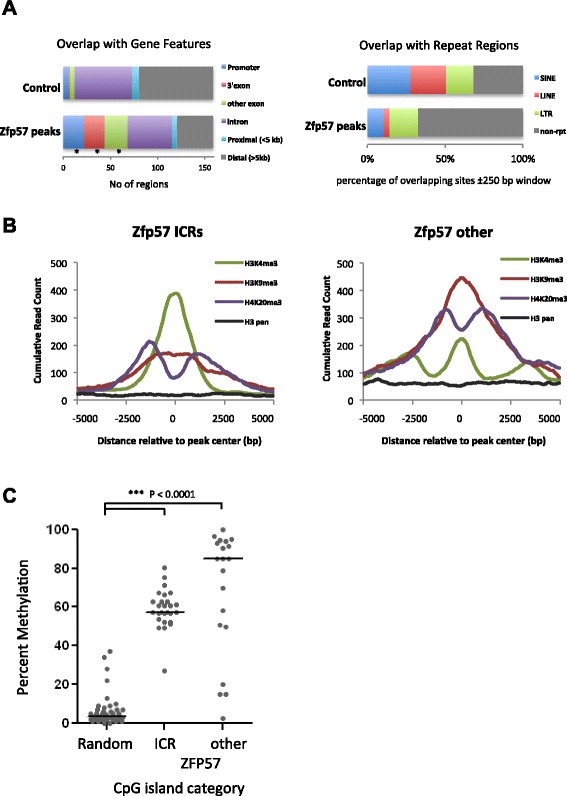
Epigenetic modifications around ZFP57 peaks*.* **a** Overlap of ZFP57 peaks with Ensembl annotated gene features (*left*) and repeat elements (*right*). Selective enrichment/depletion of ZFP57 peaks are compared against an average of ten independently generated boot-strap sets (*Control*). **b** Plot of cumulative read counts for three different histone modifications around ZFP57 peaks found at imprinted germline DMRs and elsewhere. Shown are H3K4me3, H3K9me3 and H4K20me3, and pan-H3 representing overall histone density. **c** Average DNA methylation at CGIs bound by ZFP57 at ICRs and other non-imprinted loci compared with a randomly selected set of similar size. Data points represent individual CGIs with a horizontal line showing median value for the group. Previously published ES cell reduced representation bisulfite sequencing methylation data were used for this analysis [31]. Non-parametric Kruskall-Wallis tests were performed with significantly different categories denoted (***P < 0.001)

The distribution of genomic and epigenetic features around ZFP57-bound genomic regions was analyzed compared with control unbound regions. ZFP57 peaks were significantly enriched within gene promoters (almost exclusively at known imprinted genes) and exons, including 3′ exon/untranslated regions of several zinc finger genes (Fig. 2a). Amongst repeat elements, ZFP57 peaks were depleted at SINEs (short interspersed nuclear elements) and LINEs (long interspersed nuclear elements) but not long terminal repeat (LTR) elements, including five enriched intracisternal A particles (column W in Additional file 2). This is consistent with the previously ascribed roles for its partner KAP1 in regulating zinc finger protein genes via 3′exons [26] and the silencing of endogenous retroviral elements, such as intracisternal A particles, in ES cells [27]. Our data suggest that ZFP57 is, at least in part, involved in targeting KAP1 to these elements in addition to its role in maintaining methylation imprints.

Published datasets for genome-wide epigenetic states in ES cells [28] were used to generate comparative consensus profiles for histone modifications over and around ZFP57 binding sites at both the imprinted germline DMRs and other target sites. ZFP57 targets were found to be strongly associated with both active (H3K4me3) and repressive (H3K9me3/H4K20me3) histone marks over the imprinted DMRs, but had only the repressive marks over non-imprinted regions (Fig. 2b). Given that H3K4me3 modification is normally associated with the unmethylated allele of imprinted germline DMRs [28–30] and ZFP57 targets the opposite methylated allele, we conclude that ZFP57 binding is largely associated with repressive marks, consistent with its ability to recruit the KAP1 co-repressor complex that includes the histone H3K9 methylase SETDB1. Furthermore, the shape of the profile suggests that ZFP57 acts as a nucleation site for H3K9me3 deposition and spreading that is then closely followed by appearance of the H4K20me3 mark at either side of H3K9me3.

As ZFP57 has been shown to be a DNA methylation maintenance factor both in the embryo and in ES cells [19, 21, 22], we investigated if ZFP57 peaks are generally associated with methylated regions in the genome in ES cells. We utilized available ES cell methylation data [31] and compared the methylation levels of CpG islands (CGIs) bound by ZFP57 with an equal pool of randomly selected unbound CGIs (Fig. 2c). As expected, unbound CGIs were unmethylated while ~40–60 % methylation was observed for the imprinted DMR-associated CGIs. The majority of non-imprinted CGIs bound by ZFP57 were completely or partially methylated, consistent with its methyl-sensitive binding and a role in maintaining DNA methylation. Of note, however, public datasets used for comparison were generated from serum-grown ES cells, whilst the hybrid ES cells used in our study were adapted to 2i LIF conditions. Finally, we also analyzed public reduced representation bisulfite sequencing and BS-seq datasets for germ cell and blastocyst DNA methylation [32, 33] and found that CGIs targeted by ZFP57 in ES cells are also normally methylated in the oocyte and retain methylation marks in pre-implantation blastocysts (Fig. S4 in Additional file 1).

Our DNA sequence analysis around ZFP57 bound sites has identified a heptamer TGCCGCN consensus binding motif (Fig. 4a) consistent with a hexamer described in a previous report [21] with a preference for a purine (R) rather than a pyrimidine (Y) base in the seventh base. (columns K and L in Additional file 2). This heptamer motif is often present in multiple copies. The degree to which the last base affects the ZFP57 binding affinity remains to be elucidated, however.

### Parental allele-specific Zfp57 peaks exclusively mark ICR elements

Allele-specific analysis of ZFP57 targets revealed 23 maternal and one paternal chromosome-specific instance of allele-specific ZFP57 binding (Fig. 1b), which mapped exclusively within known imprinted germline DMRs (Table 2). Figure S5 in Additional file 1 shows UCSC genome browser views for all instances of parental allele-specific binding of ZFP57 in addition to examples shown in Fig. 1b. Interestingly, these parental allele-specific peaks alone can independently define 15 of 21 known imprinted germline DMRs [33]. The other known imprinted regions were also strongly associated with ZFP57 binding, but lacked informative allele-specific reads to distinguish their parental origin (Table 2). Critically, within imprinted regions, ZFP57 targeted only germline DMRs and not secondary somatic DMRs that are established after fertilization as a consequence of the germline DMR. Furthermore, within the *Gnas* imprinted cluster, which harbors two germline-derived DMRs, ZFP57 was found to specifically associate with the one acting as the principal ICR for the region [14].

We validated parental allele-specific binding at several germline DMRs using independent ChIP assays followed by quantitative SNP pyrosequencing across BL6/Cast variant bases (Fig. S6 in Additional file 1). In addition, we experimentally identified SNPs within the two other paternally methylated (*Igf2-H19* and *Rasgrf1*) ICRs and found that our profiled ZFP57 peaks were bound on the normally methylated paternal allele (Fig. S6 in Additional file 1).

Recently it has been shown that the regional extent of methylation at the two paternally methylated ICR elements at Dlk1-Dio3 and H19-Igf2 is reduced during genome-wide demethylation in pre-implantation development stages, leaving only a core region protected from methylation reprogramming [34, 35]. The positions of ZFP57 binding coincide with the boundaries of the core protected regions in both ICRs, consistent with the role of ZFP57 in maintaining DNA methylation (Fig. S7 in Additional file 1). Interestingly we also find that most ZFP57-bound germline DMRs harbor clusters of two or more consensus motifs, suggesting that multiple ZFP57 molecules may be recruited to regions within a DMR (Table 2).

### ZFP57-bound germline DMRs identify novel imprinted genes

At least three studies have now profiled the methylomes of mouse germ cells and early embryos, identifying more than 100 novel germline DMRs that might potentially constitute novel ICRs [32, 33, 36]. Given the specificity of ZFP57 binding for ICR elements, we hypothesized that those bound by ZFP57 are most likely to be regulating genomic imprinting. Genomic regions showing differential methylation between egg and sperm (≥75 % in one germ cell type and ≤25 % in the other) and that retained ≥40 % methylation at blastocyst stage (data taken from [32, 33]) were assessed for ZFP57 binding sites. Reassuringly we could identify most known imprinted germline DMRs using this approach, including the recently reported maternally methylated germline *Gpr1* DMR on chromosome 1 (*Gpr1-Zdbf2* cluster) [33, 37, 38] and *Cdh15* DMR on chromosome 8 [39–41]. Interestingly, no Zfp57 binding was observed to the paternally methylated germline DMR in the *Gpr1*-*Zdbf2* imprinted locus, which is not the predicted ICR [42, 43]. The *Gpr1* DMR is associated with expression of a long non-coding RNA isoform of the *Zdbf2* gene (*Zdbf2linc/Liz*) that we and others find to be imprinted and paternally expressed in extra-embryonic tissues (Fig. S8 in Additional file 1) [37].

**Fig. 3:**
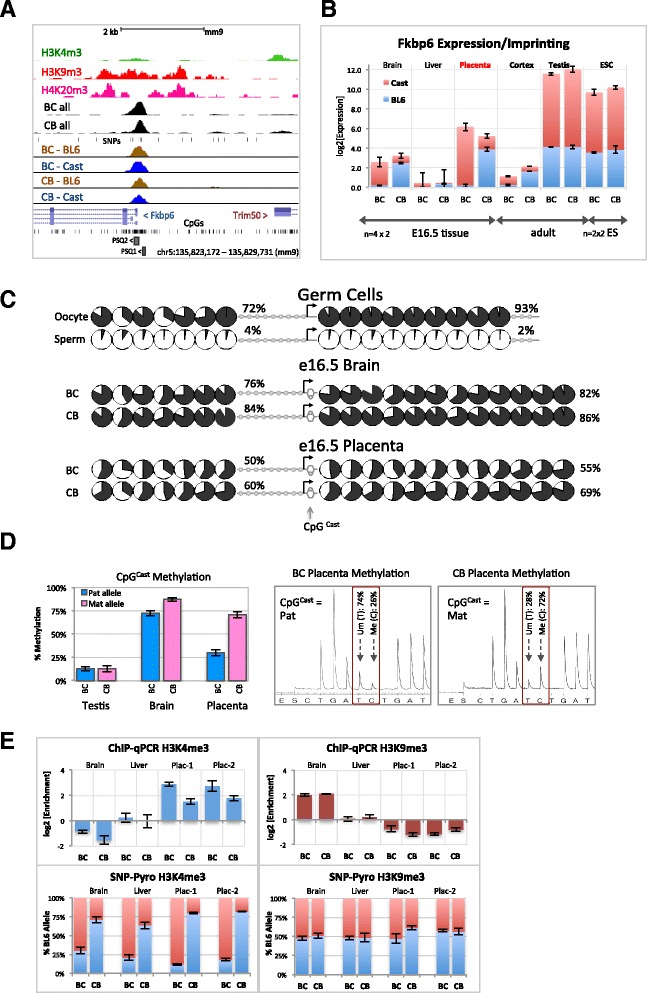
Identification of *Fkbp6* as a novel imprinted gene. **a** The *Fkbp6* gene region with the location of ZFP57 binding within the promoter regions. *PSQ1* and *PSQ2* denote regions analyzed by bisulphite pyrosequencing shown in (**c**). **b** *Fkbp6* expression levels and allele-specificity in mouse tissues and ES cells from quantitative RT-PCR and SNP pyrosequencing. The height of each bar represents Fkbp6 mRNA levels normalized to 18 s rRNA and shown relative to liver expression levels. *Blue* and *red stacked bars* show relative contribution of BL6 and Cast alleles, respectively, to overall *Fkbp6* levels. *Upper* and *lower error bars* correspond to standard deviation between biological replicates for PCR and pyrosequencing analysis, respectively (n = 8 replicates per each tissue: 4 BC and 4 CB; n = 4 for ES cell lines (*ESCs*). **c** The *Fkbp6* promoter is not a somatic DMR. Quantitative bisulphite pyrosequencing analysis of the *Fkbp6* promoter region, which assays overall methylation level in the population of cells in a non-allele-specific manner. Relative proportions of *black* (methylated) and *white* (unmethylated) filled areas in circles depict average percentage methylation for individual CpGs (n = 4; 2 BC and 2 CB bioreplicates for each tissue type). Numbers adjacent show median percentage methylation values across CpGs covered by *PSQ1* and *PSQ2* sequencing primers. *Grey circles* denote CpGs not covered in the assay. *Black arrow* denotes *Fkbp6* transcription start site and direction of expression. Grey arrow underneath indicates position of the Cast-specific CpG (SNP rs13487942), quantified in (**d**). **d** *Left*: Bar chart showing methylation status of the CpG^Cast^ when inherited on paternal (BC cross, *blue bars*) and maternal (CB cross, *pink bars*) chromosomes. Data from pyrosequencing of adult testis, E16.5 brain and placenta, n = 2 × BC and 2 × CB samples per tissue. *Right*: Representative bisulphite pyrograms from individual BC and CB placenta tissues. CpG^Cast^ region is framed in *red*, methylation level is calculated from the relative height of ‘*T*’ (unmethylated, *Um*) and ‘*C*’ (methylated, *Me*) peaks. **e** *Top*: Relative enrichment of H3K4 and H3K9 tri-methylation in E16.5 tissues. Data shown relative to input and normalized to a reference non-enriched genomic location. *Bottom*: Allele ratios from SNP pyrosequencing of ChIP-enriched DNA. Error bars reflect standard deviation between three technical replicates for each sample shown

Of the remaining novel imprinted gene candidates, we were able to design assays to test imprinting of three genes (*Nbas*, *Zfp444 and Fkbp6*), analyzing their allele-specific expression in a panel of tissues isolated from reciprocal hybrid BC and CB conceptuses at embryonic day (E)16.5. While expression of *Nbas* and *Zfp444* was biallelic in all tissues tested (data not shown), *Fkbp6* showed clear imprinted, paternal allele-specific expression in all embryonic stage tissues with highest levels observed in placenta (Fig. 3b). Highest overall expression levels were found in adult testis, consistent with a previous report [44], however, in that tissue we found expression to be biallelic. We ruled out the possibility that the observed imprinting can be a result of a technical artifact amplifying transcripts expressed at low levels using a serial dilution of testis cDNA, showing that *Fkbp6* levels in embryonic tissues are still within a linear range of amplification and that allele-specific analysis using SNP pyrosequencing is accurate at a wide range of starting concentrations (Fig. S9A in Additional file 1). We also tested whether imprinted expression extended to the neighboring *Trim50* gene, but found biallelic expression in all tissues analyzed (data not shown).

We tested if the observed imprinted expression of *Fkbp6* is associated with a constitutive DMR at the promoter. First, using allele non-discriminatory quantitative bisulphite pyrosequencing, we determined that the ZFP57 binding site in *Fkbp6* is an oocyte methylated germline DMR in vivo that is selectively maintained in placenta (~50-60 % overall methylation), but becomes hypermethylated (~80-90 % methylation) in embryo and adult somatic tissues (Fig. 3c; Fig. S9B in Additional file 1). We next determined allele-specificity of methylation using M. m. Cast polymorphism (rs13487942), which confers a Cast genome-specific CpG dinucleotide adjacent to Fkbp6 transcription start site. Consistent with the DMR status in placenta, this CpG was predominantly methylated when inherited on the maternal chromosome and hypomethylated when inherited on the paternal chromosome (Fig. 3d). Therefore, unlike most canonical ICRs, *Fkbp6* is not fully methylated or unmethylated on either of the parental alleles, perhaps indicating that only a subset of cells within the placenta have imprinting control. In addition to DNA methylation we also analyzed histone modification profiles around the *Fkbp6* promoter (Fig. 3e). Consistent with the observed expression pattern, an active H3K4me3 mark was found to be highest in the placenta and lowest in brain, whilst the opposite was observed for the repressive H3K9me3 mark. Allele-specific analysis clearly showed paternal chromosome enrichment for H3K4me3 even in brain and liver samples and was consistent with the observation of low, albeit still, imprinted *Fkbp6* expression in these tissues, demonstrating the sensitivity of this approach (Fig. 3b, e).

Interestingly, in the ES cells, the ZFP57-bound promoter peak was biallelic on a generally hypomethylated allele and the *Fkbp6* gene was robustly expressed from both chromosomes (Fig. 3a,b). Promoter methylation was comparable to that of testis (Fig. S9B in Additional file 1). We hypothesize that the loss of germline methylation memory is a result of *in vitro* ES cell culture. However, in vivo the *Fkbp6* promoter region constitutes a novel imprinted germline DMR associated with widespread imprinted expression of this gene. Tissue and/or developmental stage-specific imprinting of other candidate genes found to be biallelic in our analysis cannot be ruled out. In conclusion, our data indicate that ZFP57-bound germline DMRs serve as a good predictor of genomic imprinting.

### Genetic variation can determine monoallelic expression via ZFP57

**Fig. 4:**
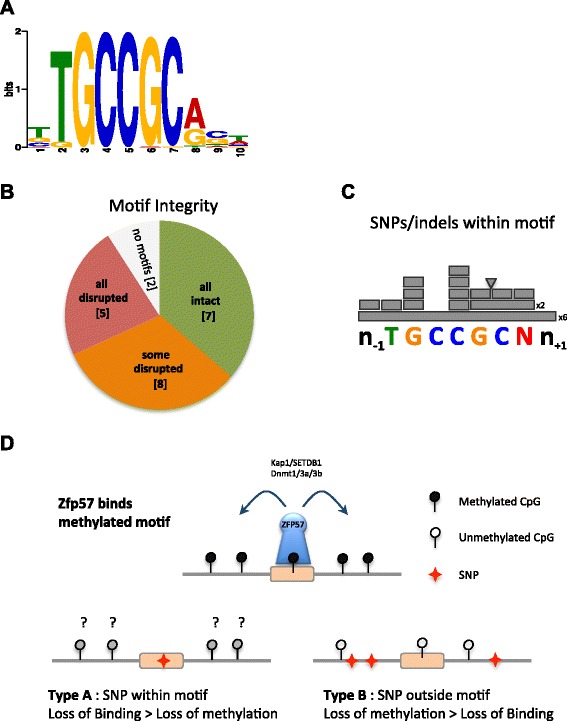
Genetic analysis of ZFP57 consensus binding motif*.* **a** Consensus ZFP57 binding motif identified using MEME algorithm analysis of DNA sequences underlying ChIP-seq peaks. **b** Distribution of genetically determined monoallelic Zfp57 peaks based on motif integrity on the unbound vs. bound alleles: SNPs disrupting all of the motifs (five sites, *red*), some of the motifs with at least one remaining intact (eight sites, *orange*) and none of the motifs (seven sites, *green*). No motifs could be found on either genetic alleles in two cases (*grey*). **c** Diagram indicating which bases within the consensus are varied between BL6 and Cast genomes in strain-specific peaks. *Long bars*, *short bars* and *triangle* indicate deletion, SNP and insertion events, respectively. **d** Putative mechanisms for ZFP57 allele-specific binding. *Top*: ZFP57 bound, motif intact and methylated. *Bottom left*: ZFP57 does not bind because motif is mutated. *Bottom right*: ZFP57 does not bind because motif is unmethylated in a given genetic background

Analysis of allele-specific ZFP57 binding revealed a subset of monoallelic peaks that were associated with either the BL6 (17) or Cast (9) allele regardless of parental origin (Fig. 1b). We hypothesized that these might have arisen through strain-specific genetic variation in the binding motifs (Fig. 4a). We successfully sequenced 22 out of 26 ZFP57-bound regions from pure C57BL/6 and Cast/EiJ animals and identified intact and disrupted motifs present on bound vs. unbound alleles for each peak (Additional file 3). Individual motifs were considered to be disrupted even if a single base pair change occurred anywhere within the heptamer motif sequence (Additional file 4) [21, 45]. Given that many peaks contain multiple motif instances, we categorized each site as follows: all motifs disrupted (no intact motifs on unbound allele); some motifs disrupted (at least one intact motif remaining on unbound allele); and all motifs intact despite apparent monoallelic binding (Fig. 4b). Surprisingly, we identified only five peaks with all motifs disrupted. Eight regions had only some motifs disrupted and in seven regions the genetic variation did not affect the motif sequence at all, but was located outside adjacent to it (Fig. 4b).

Examination of the motif-disrupting SNP types indicated that the majority were single base substitutions found in different positions of the motif with the highest frequency located over the central CpG dinucleotide. Other disruptions included deletions of the entire motif or part thereof and, in one instance, a 3 bp insertion within the motif (Fig. 4c).

**Fig. 5:**
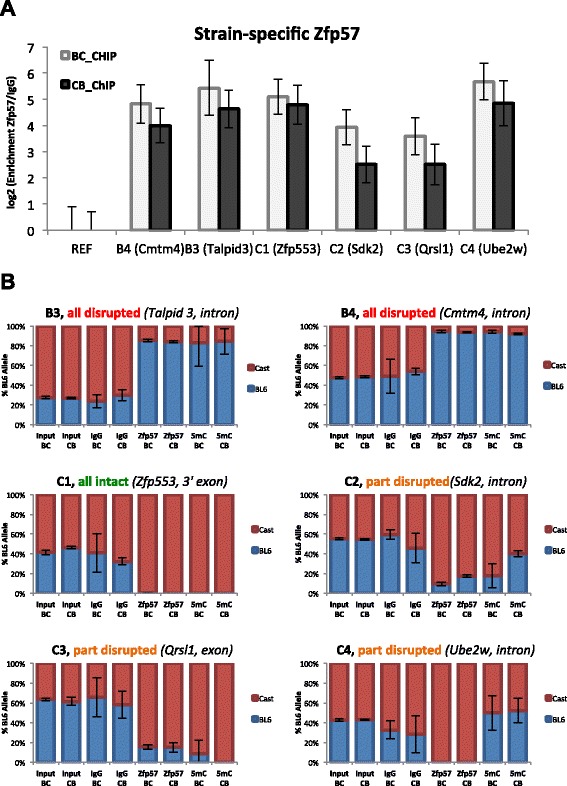
Validation of strain-specific sites*.* **a** Independent ChIP-quantitative PCR validation of Zfp57 binding to non-imprinted monoallelic peaks. Overall enrichment levels are shown relative to negative control region (*REF*) and normalized to non-specific IgG pull-down. **b** Allele-specific analysis using SNP pyrosequencing of ChIP and 5mC-DIP enriched DNA showing Zfp57 binding is directed by both underlying DNA sequence and methylation status of the allele. Error bars represent standard deviation between three technical replicates

Given that, in multiple cases, SNPs within the motif sequence alone could not explain the strain-specific ZFP57 binding, we predicted that genetic variation outside the motif might determine the methylation state of the motif and in turn explain strain-specific binding at these sites (Fig. 4d). To test this we conducted parallel independent ZFP57-ChIP and methylated DNA immunoprecipitation (MeDIP) assays (Fig. 5) followed by SNP pyrosequencing of precipitated regions. The ratio of BL6 to Cast alleles was quantified and in each case we were able to confirm strain-specific ZFP57 binding. In five of six cases tested the region was also associated with differential methylation. This included a ZFP57 peak C1 at the 3′ end of *Zfp553* (Fig. 5b, left middle chart), which had intact motifs on both alleles but was associated with DNA methylation and ZFP57 binding specifically on the Castaneus sequence. We suggest that the strain-specific differential methylation in these instances may be genetically conferred, perhaps by neighboring genetic variants.

**Fig. 6:**
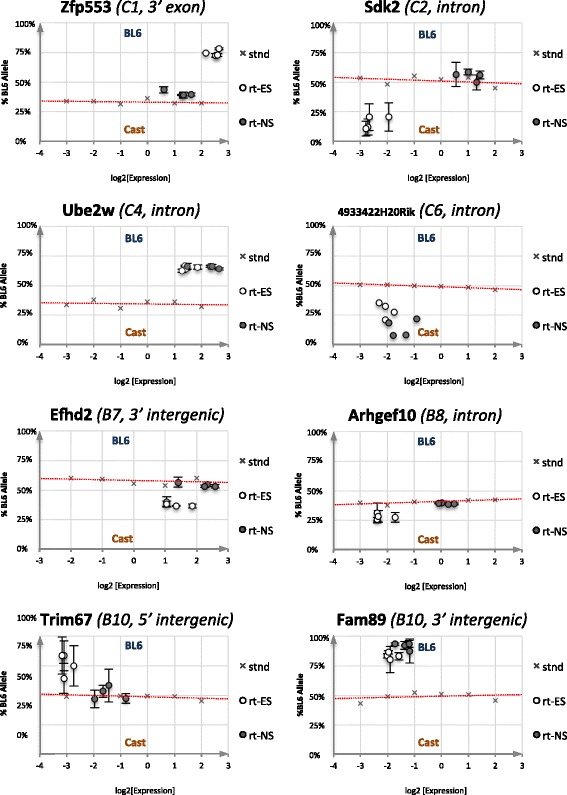
Strain-specific expression bias at genes around non-imprinted Zfp57 peaks. Allelic activity and expression level for eight genes found to have significant allelic bias in hybrid ES cells (*ES*, *clear dots*) and their neural stem cell derivatives (*NS*, *filled dots*); see also Fig. S10 in Additional file 1 and Table 3. The corresponding genomic DNA standard (expected 50 % allele composition) used to control for PCR bias and pyrosequencing artifacts is shown as a fitted linear regression (*red dotted line*). Allelic composition found by SNP pyrosequencing is plotted along the y-axes as percentage of BL6 expression (0 % = pure Cast, 100 % = pure BL6 and 50 % = perfect biallelic) and overall expression level by quantitative RT-PCR is plotted along the x-axes as absolute levels of the control hybrid genomic DNA (log2 nanograms/PCR). The title in each graph shows gene name and closest associated ZFP57 peak along with its relative position with respect to the gene. Note that for *Sdk2* a dilution series of neural stem cell cDNA (high level biallelic expression) was used as a control since the relevant exon boundary panning primers could not amplify genomic DNA

We next determined whether the strain-specific genetically determined monoallelic binding, could be associated with the expression of 19 genes adjacent to these sites. Allele-specific expression was investigated using four different reciprocal hybrid ES cell lines and neural stem (NS) cell progenitors derived from them. Data identified eight genes in ES cells with significant strain-specific allele expression bias (Fig. 6, Table 3; Fig. S10 in Additional file 1). Four of these (*Efhd2*, *Arhgef10*, *Trim67* and *Sdk2*) were upregulated and became biallelic upon neural differentiation. Three (*Ube2w*, *Fam89a* and *4933422H20Rik*) retained allele bias upon differentiation and one (*Zfp553*) was downregulated and became biallelic in NS cells. The remaining genes were equally expressed from both alleles or showed minimal skews (<15 % median skew relative to genomic DNA control; Table 3; Fig. S10 in Additional file 1). There was no obvious pattern relating the position of the ZFP57-bound region and the relative allelic activity. For example, while Zfp57 binding at the Cast allele of the *Zfp553* gene was associated with BL6-specific expression, the *Sdk2* gene showed preferential expression from the ZFP57-bound Cast allele.

**Table 3:** Relative allele usage in expression of genes neighboring strain-specific ZFP57 peaks

		Genomic DNA				cDNA ES cells					cDNA NS cells				
Gene	Zfp57 peak	BC2	BC8	CB4	CB9	BC2	BC8	CB4	CB9	ANOVA	BC2	BC8	CB4	CB9	ANOVA
B2_Nhlrc1	3′ intergn^a^	38 %	35 %	32 %	38 %	28 %	21 %	38 %	39 %	NS	-	-	-	-	-
B3_Talpid3	Intron	51 %	52 %	51 %	50 %	51 %	51 %	51 %	51 %	NS	54 %	52 %	52 %	53 %	*
B4_Cmtm4	Intron	49 %	52 %	49 %	48 %	54 %	55 %	52 %	52 %	*	47 %	50 %	47 %	49 %	NS
B7_Efhd2	3′ intergn	52 %	53 %	59 %	56 %	**37 %**	**37 %**	**40 %**	**38 %**	***	53 %	55 %	53 %	57 %	NS
B8_Arhgef10	Intron	42 %	45 %	41 %	40 %	**31 %**	**27 %**	**26 %**	**28 %**	***	40 %	39 %	39 %	40 %	NS
B10_Fam89	3′ intergn	52 %	48 %	47 %	47 %	**84 %**	**83 %**	**80 %**	**87 %**	***	**93 %**	**92 %**	**94 %**	**88 %**	***
B10_Trim67	5′ intergn	45 %	48 %	45 %	46 %	**64 %**	**75 %**	**84 %**	**84 %**	**	47 %	53 %	47 %	58 %	NS
B12_Nbas	Intron	53 %	44 %	54 %	54 %	66 %	48 %	65 %	66 %	NS	53 %	44 %	54 %	54 %	NS
B13_Ankrd6	Intron	49 %	49 %	47 %	51 %	52 %	51 %	54 %	55 %	NS	41 %	43 %	37 %	44 %	**
B15_Btbd11	Intron	58 %	59 %	59 %	57 %	56 %	57 %	57 %	52 %	NS	53 %	54 %	57 %	57 %	NS
B16_Ccdc11	5′ intergn	51 %	51 %	52 %	53 %	62 %	71 %	62 %	66 %	*	62 %	60 %	62 %	47 %	NS
C1_Zfp553	3′ exon	36 %	35 %	36 %	34 %	**73 %**	**78 %**	**73 %**	**75 %**	***	39 %	39 %	39 %	43 %	*
C2_Sdk2	Intron	-	-	-	-	**12 %**	**11 %**	**20 %**	**20 %**	**	59 %	56 %	50 %	56 %	NS
C3_Qrsl1	Exon	50 %	46 %	45 %	48 %	46 %	49 %	51 %	49 %	NS	47 %	48 %	50 %	49 %	NS
C4_Ube2w	Intron	40 %	37 %	39 %	38 %	**67 %**	**66 %**	**64 %**	**63 %**	***	**66 %**	**66 %**	**64 %**	**66 %**	***
C5_Usp14	Intron	47 %	45 %	46 %	47 %	47 %	45 %	45 %	46 %	NS	43 %	42 %	43 %	43 %	*
C6_4933422H20Rik	Intron	50 %	48 %	46 %	49 %	**21 %**	**28 %**	**32 %**	**36 %**	**	**8 %**	**19 %**	**8 %**	**22 %**	***
C7_Tmem86a	3′ exon	52 %	49 %	52 %	52 %	52 %	41 %	51 %	59 %	NS	46 %	45 %	48 %	44 %	NS
C8_Atrn	Intron	41 %	37 %	37 %	37 %	45 %	42 %	43 %	47 %	*	36 %	38 %	40 %	46 %	NS

^a^ *Intergn* intergenic ZFP57 binding upstream (5′) or downstream (3′) of the gene. Each row shows the gene name, position of the associated ZFP57 binding site and percentage of BL6 allelic expression ascertained by SNP pyrosequencing (i.e., 100 % = exclusively BL6, 0 % = exclusively Cast and 50 % = perfect biallelic expression). cDNA from four hybrid ES and neural stem cells was analyzed and compared with their corresponding genomic control for PCR bias (gDNA). Values in bold font emphasize significant C57BL/6 or Cast preferential allelic expression (*p* < 0.01, median difference greater than 15 % compared with genomic DNA control). For *Sdk2*, where no gDNA is available, ES cells are compared relative to neural stem cell expression. (*p < 0.05, **p < 0.01, ***p < 0.001 repeated measures ANOVA with Bonferroni’s post-test; *NS* not significant). See also Fig. S10 in Additional file 1 and Fig. 6 highlighting the relative expression level and allelic preference for eight highlighted genes.

## Discussion

We and others have recently focused on the role of ZFP57 and KAP1 in the maintenance of DNA methylation in early mouse embryo development [19, 21–23]. In the current work we extend these studies by performing parental-origin allele-specific analysis of endogenous ZFP57 binding using reciprocal hybrids. Critically, unlike previous studies, we distinguish monoallelic binding determined by parental origin effects (imprinted germline DMRs) from those determined by underlying sequence variation.

We identified ZFP57 binding within all known imprinted regions, where it specifically targets the methylated allele of germline but not somatically methylated DMRs. In fact all parental allele-specific peaks lie exclusively within well-established or recently reported imprinted germline DMRs. Most ZFP57 sites are associated with peaks of repressive H3K9 and H4K20 tri-methylation, and recruitment of KAP1 and associated SETDB1 H3K9-specific histone methyltransferase to these regions. In addition, peaks of active H3K4me3 modification were present solely at the imprinted germline DMRs, where they are associated with the opposite unmethylated allele, consistent with these three marks being hallmarks of ICRs in ES cells [29, 46]. A larger proportion of ZFP57 peaks are, however, outside imprinting clusters and, in particular, found within gene exons and introns and frequently overlap LTRs corresponding to endogenous retroviral (ERV) insertions (columns V and W in Additional file 2). These are targeted by KAP1, which is necessary to silence ERVs in mouse ES cells [26, 27, 47], suggesting that silencing at these loci is targeted, at least in part, by ZFP57.

ZFP57 occupancy at identified genomic location, however, does not constitute a biochemical assay of function. For instance, whilst we and Quenneville et al. [21] observe ZFP57 binding to all known germline ICRs, only a subset of these is hypomethylated in the Zfp57 null embryos even in maternal zygotic mutants [19]. This might be explained by redundancy mediated by other KRAB-ZFPs that could potentially recruit KAP1 to the unaffected ICRs or through action of other factors such as PGC7/Stella [48]. Alternatively, in the absence of ZFP57 binding, kinetics of DNA methylation loss may vary between different ICRs. As there is only a finite window of pre-implantation development during which the global DNA demethylation wave could potentially target imprints, some ICRs may retain sufficient methylation to allow recovery. Indeed at the *H19* locus there is evidence for stochastic loss of methylation in embryos derived from KAP1 null oocytes [23, 49]. Finally, loss of DNA methylation may be secondary to loss of histone H3K9 tri-methylation in the absence of KAP1-SETDB1 recruitment to ICRs. Levels of H3K9me3 at ICRs has not been analyzed in pre-implantation embryos.

There is considerable interest in determining the full imprinted complement of the mouse genome and in identifying novel imprinted genes. Given that all murine imprinted clusters identified to date contain a germline DMR, one can also survey the DNA methylation in egg, sperm and early embryos to identify candidate imprinted loci. Based on data from two such studies, approximately 1000 CGIs are hypermethylated in mature oocyte and hypomethylated in sperm, of which only ~15 % retain methylation in the blastocyst [32, 33]. This still provides over 100 novel putative candidate imprinted clusters. We hypothesized that those bound by ZFP57 may represent *bona fide* ICRs for novel imprinted genes. Overlapping ZFP57 peaks with these extended germline DMRs identified eight potential candidates, of which three had already been recently analyzed and *Cdh15* reported as imprinted [40]. We tested additional three and report identification of another novel imprinted gene, FK506-binding protein 6 (*Fkbp6*) on chromosome 5. Previously, Fkbp6 has been identified as a testis-specific factor of the meiotic synaptonemal complex, with mutant male but not female mice being completely sterile [44]. Another study has also implicated *Fkbp6* in the piRNA biogenesis pathway in male germ cells [50]. *Fkbp6* expression is abundant and not imprinted in adult testis, presumably as a result of imprint reprogramming in the germline (Fig. 3b). In embryonic tissues it is widely imprinted, with the most robust expression being in the placenta. Further experiments are necessary to uncover the functional relevance of *Fkbp6* imprinting in placenta tissues.

In human patients with Williams syndrome carrying a large multi-gene deletion on chromosome 7q11.23, *FKBP6* hemizygocity was found to contribute to hypercalcemia and growth retardation [51]. Growth retardation in Williams syndrome has been associated with a parental-origin effect [52]. Given recent reports that human ZFP57 recognizes the same DNA consensus motif [53], we analyzed the promoter sequence of the human *FKBP6* gene and identified at least two putative ZFP57 binding motifs. ENCODE data suggest that the human promoter CGI is methylated in most normal and cancer human cell lines as well as adult brain tissue (Fig. S11 in Additional file 1). Hence, akin to mouse, imprinting of the human *FKBP6*, if it occurs, is likely to be tissue specific.

It has been demonstrated in vitro that ZFP57 zinc fingers 2–3 are responsible for binding motif recognition and have the highest affinity when a central CpG dinucleotide in the motif is fully methylated [21, 45]. Here we demonstrate this in vivo for the endogenous full-length protein, showing that ZFP57 always follows the methylated allele in cases of both imprinted (Fig. S6 in Additional file 1) and genetically determined allele-specific binding sites (Fig. 5). We also identify the same consensus binding motif sequence as Quenneville et al. [21], noting a clear preference for purine (R) over pyrimidine (Y) as the last base of the heptamer motif (columns K and L in Additional file 2). We note that in case of guanine (around half of all motifs found) this creates a second CpG site, which we often find to be concomitantly methylated with the central CpG in the motif. It remains to be experimentally elucidated, however, whether ZFP57 binds with higher affinity to the sequence TGCCGCR than TGCCGCY.

In situations where binding was abrogated in a strain-specific manner, defects in the binding motif preferentially fell within that central CpG. In monoallelic strain-specific binding where the motif(s) itself is genetically identical on both alleles, ZFP57 was again targeted to the methylated allele (Fig. 5). In these instances, however, adjacent DNA polymorphisms were noted. Genetic variation in *cis* has previously been shown to influence CG methylation in mouse and human and hence provides a likely explanation for this differential methylation [54–57].

Much attention has focused on understanding the mechanisms by which genetic variation can determine divergence in gene expression levels. Expression quantitative trait loci (eQTL) studies have shown that a large proportion of genes can be influenced by particular genetic variants [58]. A recent study found that approximately a quarter of genes expressed in mouse liver show strain-specific differences in expression between C57BL/6 and Cast/EiJ mice [59]. In our study we find a large proportion of ZFP57 sites being targeted to either BL6 or Cast chromosomes regardless of parental origin. We tested whether genes in the vicinity of these sites exhibit skewed allelic expression and find that more than 40 % (8/19) of the genes tested show significant preference of one allele over the other in BC and CB hybrid ES cells (Fig. 6; Fig. S10 in Additional file 1). Upon differentiation into the neural lineage, *Zfp57* mRNA levels are dramatically reduced and half of these genes become biallelically expressed, whilst the other half remain expressed in a predominantly monoallelic fashion. Interestingly, ZFP57 binding and gene expression were not always inversely correlated as would be predicted by recruitment of a repressive KAP1 complex. Further analysis will determine whether ZFP57 is directly responsible for the allelic bias in these genes.

## Conclusions

In the current study we present four novel findings. First, we show that within 22 established mouse imprinted clusters, ZFP57 specifically binds known or predicted ICR elements in each of them, but does not interact with secondary somatically methylated DMRs or germline DMRs that are not imprinting controlling. Second, we show that ZFP57 binding can be efficiently used to predict novel imprinted genes, identifying *Fkbp6* as a gene whose expression is imprinted and paternal allele-specific in placenta. Third, we show that many ZFP57 targets lie outside imprinted clusters, have biallelic binding, are hypermethylated and are enriched in repressive H3K9me3 histone marks, consistent with KAP1 co-repressor recruitment; members of this class include some ERVs where the DNA binding factor targeting the repressive state had not previously been identified. Finally, we show that genetic and epigenetic variation can specify strain-specific monoallelic ZFP57 binding, which is often associated with biased allelic expression of adjacent genes.

## Materials and methods

### Cell culture

Reciprocal hybrid mouse ES cells were derived in serum replacement media as described previously [60] and adapted to feeder-free-based 2i LIF culture conditions (N2B27, Stem Cell Sciences) [61]. Monolayer neural differentiation was induced by withdrawal of 2i and LIF supplements using established protocols [62, 63]. Derived neural stem cells were maintained in RHB-A media (Stem Cell Sciences) supplemented with10 ng/ml of FGF-2 and EGF (Peprotech). Research was conducted in accordance with UK Home Office Animals Scientific Procedures Act, project licence 80/2567.

### Western blotting

Whole cell lysates prepared in 1× Laemmli buffer (10 μg) were loaded in each well of a 10 % acrylamide gel. Following transfer the blots were probed with rabbit anti-Zfp57 (Abcam, ab45341) at 1:5000 dilution or mouse anti-Tubulin (SIGMA, T6199) at 1:10,000 dilution.

### ChIP analysis

ChIP analysis of Zfp57 binding was performed on formaldehyde cross-linked chromatin isolated from ten 10-cm dishes of hybrid mouse ES cells grown to ~70 % confluency. Briefly, formaldehyde was added directly to cell media to a final concentration of 1 % and dishes were incubated at room temperature for 10 minutes with agitation. The reaction was stopped by addition of glycine to a final concentration of 0.125 M. The cells were washed twice in ice-cold phosphate-buffered saline (PBS), scraped into falcon tubes, centrifuged and resuspended in cell lysis buffer (0.25 % Triton X-100, 10 mM EDTA, 0.5 mM EGTA, 10 mM Tris pH 8.0). Isolated nuclei were lysed in sonication buffer (50 mM Tris-HCl pH 8.0, 10 mM EDTA, 0.5 % SDS) and sheared to an average size of 200–500 bp using a Bioruptor sonicator (Diagenode, UCD-200). Chromatin from ~5 × 10^6^ cells was diluted five-fold with IP buffer (1.1 % TX-100, 1.2 mM EDTA, 16.7 mM Tris pH 8.1, 167 mM NaCl) and pre-cleared with 10 μg non-immune rabbit IgG and 50 μl of protein A magnetic beads (Dynabeads, Invitrogen) for 3 h at 4 °C on a rotating wheel. Anti-Zfp57 antibody (4 μg, abcam ab45341) or normal rabbit IgG was added to pre-cleared chromatin and incubated overnight at 4 °C on a rotating wheel. Chromatin was precipitated with 25 μl protein A beads for 3 h at 4 °C with rotation. The beads were then washed in each of the following: buffer 1 (1 % Triton X-100, 0.1 % SDS, 2 mM EDTA, 150 mM NaCl and 20 mM Tris pH 8), buffer 2 (1 % Triton X-100, 0.1 % SDS, 2 mM EDTA, 500 mM NaCl and 20 mM Tris pH 8), buffer 3 (0.25 M LiCl, 1 % NP-40, 0.5 % sodium deoxycholate, 1 mM EDTA, 10 mM Tris pH 8) and twice with TE buffer (10 mM Tris pH 8, 1 mM EDTA). The bound chromatin was eluted into elution buffer (1 % SDS, 0.1 M NaHCO_3_), followed by crosslink reversal and protein digestion. DNA was extracted by phenol/chloroform and ethanol precipitated in the presence of 10 μg of glycogen. One-tenth of the ChIP reaction was used for quantitative PCR and SNP pyrosequencing analysis to control for ChIP efficiency. The remainder was used for Illumina library preparation.

Histone modifications were analyzed using native non-crosslinking ChIP done on di- and tri- nucleosomal preparations as described previously [64]. Mouse embryo brain, liver and placenta tissues at E16.5 (F1 BL6/Cast reciprocal hybrids) were analyzed using the following antibodies: anti-H3K4me3 (Diagenode, C15410003); anti-H3K9me3 (Abcam, ab8898) and anti-panH3 (control Abcam, ab1791).

### Illumina library preparation and sequencing

Sequencing libraries were prepared from ~10 ng of ChIP and 1 μg of input DNA using NEBnext® kit (E6260, NEB) according to the manufacturer’s protocol. The PCR amplified library was purified using Agencourt AMPure magnetic beads for gel fragments size selected at 150–400 bp. Each ChIP and input library was sequenced from a single end to a length of 40 nucleotides on individual lanes of an Illumina Genome Analyzer IIx. Allele-specific read alignment and detailed bioinformatics analysis of data are outlined in Additional file 4.

### Independent ChIP-quantitative PCR validation

To validate ChIP-seq peaks, we performed anti-ZFP57 ChIP experiments in independently grown ES cells used for ChIP-seq (BC8 and CB9; Fig. S1 in Additional file 1) as well as two additional reciprocal hybrid lines, BC2 and CB4. Pull-downs using non-immune rabbit IgG were used to control for non-specific enrichments. ChIP enriched DNA was analyzed in triplicate real time quantitative PCR reactions using SYBR-green chemistry (LC480 instrument, Roche). The comparative Ct method was used to calculate fold enrichment levels normalizing to input DNA and non-specific IgG. In order to assess statistically significant enrichment levels, we designed primers for six locations not associated with the ZFP57 peak in the ChIP-seq data. Peaks having enrichment levels three standard deviations above the mean of the negative control were marked as confirmed. Primer sequences for each tested location are listed in Additional file 5. Primers for quantitative PCR and SNP pyrosequencing analysis were designed using primer3 software [65] and Pyromark Assay Design software (Qiagen). In the case of the latter, one of the oligos was 5′ biotinylated to allow downstream processing (see below).

### MeDIP analysis

Total genomic DNA was isolated from exponentially growing ES cells. The cells were trypsinized, washed in PBS, lysed in tail buffer (50 mM Tris, pH 8.0, 100 mM EDTA, 100 mM NaCl, 1 % SDS) and incubated first with RNase A for 10 minutes at 37 °C and then Proteinase K overnight at 55 °C. DNA was extracted sequentially with phenol, phenol-chloroform-isoamyl alcohol, chloroform and then ethanol precipitated. For MeDIP, 1 μg of DNA was sheared to an average size of 200–800 bp using a Bioruptor sonicator (UCD-200, Diagenode) and incubated overnight with 2 μg of anti-5methyl-cytosine antibodies (Diagenode). The complexes were precipitated using sheep anti-mouse IgG conjugated magnetic beads (Invitrogen). The beads were washed three times with binding buffer and eluted with proteinase K. Purified DNA was subsequently PCR amplified using primers listed in Additional file 5 and analyzed by quantitative SNP pyrosequencing to determine the relative ratio of each allele.

### Reverse transcription and quantitative PCR assays

Total RNA was isolated using the TRI-reagent method from either cultured cells or homogenized tissue, DNaseI treated and converted to cDNA using a RevertAid first strand cDNA synthesis kit and random hexamer primers (Fermentas). Quantitative PCR was performed using SYBR-green chemistry on the Light Cycler 480 instrument (Roche). Three housekeeping genes (18S rRNA, β-Actin and GAPDH) were used as a control. At least two biological and three technical PCR replicates were performed.

### Pyrosequencing

Pyrosequencing was performed on a Pyromark Q96 MD instrument and PyroMark Gold Q96 Reagents (Qiagen) using the kit manufacturer's protocols and as described previously [60]. Two types of assays were performed. First, SNP pyrosequencing was used to measure the relative allele ratio following RT-PCR (monoallelic gene expression assessment) or following ChIP/MeDIP-quantitative PCR measuring allele-specific enrichment of Zfp57 or 5-methyl cytosine at given genomic locations. Primer sequences are listed in Additional file 5.

Second, quantitative bisulphite CpG pyrosequencing was performed in order to assess DNA methylation levels at the two novel germline DMRs in germ cells and E16.5 conceptus tissues. Briefly, purified genomic DNA was bisulphite converted using an imprint® DNA modification kit (SIGMA), PCR amplified using *Gpr1* and *Fkpb6* DMR-specific primers, and pyrosequenced on the PSQ HS96 system (Qiagen) using pyromark CpG analysis software. PCR product preparation was performed in the same way as for SNP pyrosequencing. The following primer sets were used:

Gpr1 DMR: forward, AGGGTTATATTGAGAGAAATATTGTG; reverse (5’ biotin), ATATTAAATTAAACCCTAAATTCCATTTCT; sequencing, ATTTAAATTATTGATGTTTAAG. Sequence to analyze (CpG positions assayed in bold): **Y**GTATTTATTGT**Y**GT**Y**GTTTTG**Y**GGTTTTGT**Y**GTTTTTATAGATGTTTTATTTTAT**Y**G**Y**GGTAT**Y**GTA**Y**GGTA**Y**GTTTTTG.

*Fkbp6* promoter DMR: forward, GTTTTGTTAGAAGTTTTTTTAGGGTTTTAT; reverse (5’ biotin), CTAACCTAAAATACCAACCCCTTCC; sequencing PSQ1, AGTTTTTTTAGGGTTTTATTTTG; sequencing PSQ2, GGGTAGTTTTAGGTAAGGTTTT. Sequence to analyze, (CpGs in bold, BL6/Cast SNP *rs13487942* in italics): PSQ1, TTTTG**Y**GTTTGTTATTA**Y**GTGTGTAG**Y**G**Y**GTGTTGGGAGATTTTTAG**Y**GTATGTT**Y**GTATGTT**Y**GTTG; PSQ2, TGG*A*/[*T*/*C***]**GATAGTTGTG**Y**GGTAAGATGAG**Y**GTTTTTT**Y**G**Y**GTTTTAGGAA**Y**GGAATTTTAT**Y**GT**Y**G**Y**GAGA**Y**GATTGTTAGGTA**Y**GGAG**Y**GGGGTT**Y**GGG.

The *Fkbp6* promoter region was amplified using the above forward and reverse primers and pyrosequenced sequentially using two sequencing primes. Genomic positions of CpGs covered by PSQ1 and PSQ2 sequencing primers are indicated in Fig. 3a (bottom tracks).

Note, quantitative bisulphite pyrosequencing is a bulk population assay measuring overall methylation levels and as such is not allele-discriminatory. We could, however, determine allele-specificity for one CpG, which was present only in M. m. Cast genome (rs13487942). We initially attempted conventional clonal bisulphite sequencing but encountered strong cloning bias towards methylated alleles even in generally hypomethylated tissues (testis and ESCs).

### Analysis of monoallelic gene expression near strain-specific ZFP57 binding sites

Assessment of allele specificity of gene expression was performed using quantitative RT-PCR followed by SNP pyrosequencing of the resulting product. The assays were performed using cDNA from four hybrid ES cell lines (2 BC and 2 CB) and neural stem cells derived therefrom. Genomic DNA corresponding to each cell line was used as a reference for 50 %:50 % BL6/Cast allele percentage relative to which skews in cDNA were analyzed (Table 3; Fig. S10A in Additional file 1). A dilution series of the reference hybrid genomic DNA was constructed to control for PCR amplification and pyrosequencing biases at a wide range of starting concentrations (2–0.25 ng/PCR).

Absolute expression level for each gene was quantified against the reference genomic DNA standard and is represented as log2 (nanograms of standard/PCR) along the x-axes in Fig. 6. Typically, 1 μl of a 1/20 cDNA dilution of the 1 μg RNA reverse transcription reaction were used per PCR. Repeated measures ANOVA was used to compare median allele ratios in cDNA relative to genomic DNA controls (Table 3; Fig. S10 in Additional file 1). An arbitrary cutoff was set for genes having *p* < 0.01 (Bonferroni post-test) and an overall difference in median allele ratio ≥15 % (either towards BL6 or Cast) relative to genomic DNA (eight genes shown in Fig. 6).

In the case of allele-specific analysis of *Fkbp6* and *Sdk2* genes, due to position of informative SNPs, PCR primers had to span exon boundaries and a reference hybrid gDNA standard could not be used to test for bias. Instead we constructed standards using a dilution series of a cDNA where we found these genes to be highly abundant and biallelic (e.g., testis for *Fkbp6* and neural stem cells for *Sdk2*). Using this control we were able to demonstrate that monoallelic expression (imprinted or genetically determined) did not arise from random PCR amplification biases.

### Data availability

Illumina sequencing data have been submitted to the Gene Expression Omnibus database under accession GSE55382.

## Additional files

Additional file 1:
**Supplementary Figures S1–S11.**
Additional file 2: Table S1. Zfp57 binding sites and their genomic annotations. Additional file 3:
**Genomic sequence alignments of C57BL/6 and Cast/EiJ DNA around monoallleic Zfp57 binding sites.**
Additional file 4:
**Supplementary methods.**
Additional file 5: Table S2. Primer sequences used in the study.

## Abbreviations

ASAP: allele-specific alignment pipeline
CGI: CpG island
ChIP: chromatin immunoprecipitation
DMR: differentially methylated region
ERV: endogenous retroviral
ES: embryonic stem cell
NS: neural stem cell
ICR: imprinting control region
LTR: long terminal repeat
MeDIP: methylated DNA immunoprecipitation
SNP: single nucleotide polymorphism
