# Supplementary material for: Allele-specific binding of ZFP57 in the epigenetic regulation of imprinted and non-imprinted monoallelic expression
Source: Genome Biol. 2015 May 30;16(1):112. doi: 10.1186/s13059-015-0672-7 (PMC4491874; doi:10.1186/s13059-015-0672-7)
Supplement: Additional file 1: — Supplementary Figures S1–S11. [file 13059_2015_672_MOESM1_ESM.pdf]

Additional File1

Supplementary Figures

Figure S1. Zfp57 expression in hybrid ES cells. Western blot analysis of ZFP57 expression in whole cell extracts isolated from several hybrid and the E14 ES cell lines. Asterisks denote cell lines used for ChIP-seq.

Figure S2. Analysis strategy for allele-specific Zfp57 binding site mapping. Schematic diagram of the short read data analysis pipeline employed to identify allele-specific binding events for ZFP57. Reads were binned into common and allele-specific alignments using ASAP algorithm (see methods). Identification of the total number of binding sites, quantification of allele-specific reads under each peak and analysis of statistically significant mono-allelic binding events were performed using MACS, Seqmonk and Fishers' exact test respectively.

Figure S3. Selection of 158 ZFP57 peaks. (A) Venn diagram showing the degree of overlap between MACS peaks identified in BC and CB hybrid ES ChIP-seq experiments (left) and the same for the randomized sets of sites of equal size (right). Commonly enriched genomic locations with combined mean read density ≥5 (55273 peaks) were taken for further analysis. (B) Ninety two peaks selected for independent ChIP-qPCR validation arranged in the order of decreasing rank (see also Additional file 2: Table S1). Confirmed and false positives are shown in green and red respectively. (C) Correlation between read density in BC vs. CB ChIP-seq experiments. Top 1 % ranking sites selected based on qPCR analysis are shown in green.

Figure S4. DNA methylation status of ZFP57 bound CGIs in germ cells and early embryo. Dot-plot diagram showing pairwise comparisons of CGI methylation found to bind ZFP57 in our ChIP-seq experiment vs. comparable number of randomly selected CGIs. Non-parametric Kruskall-Wallis tests were performed with significantly different categories denoted (***P<0.001, **P<0.01, ns - not significant). Publically available data for DNA methylation in GV oocyte, sperm and blastocyst stage embryos were used for this analysis [32].

Figure S5. Genome browser views of all instances of parental allele-specific ZFP57 binding. Each panel shows a region of genome where parental allele specific ZFP57 binding was identified. Individual track arrangement is identical to Fig. 2.

Figure S6. ZFP57 binding and parental origin specific DNA methylation analysis at imprinted gDMRs by independent ChIP and MeDIP. (A) Independent ChIP-qPCR result showing enrichment of ZFP57 at the methylated allele of several imprinted somatic and germline DMRs. (B) Quantitative SNP-pyrosequencing assay showing relative allele enrichment (shown as percentage BL6 allele, 0 % = pure Cast, 100 % = pure BL6, 50 % = perfect biallelic) in the PCR product amplified from input DNA, normal IgG pull-down, ZFP57-ChIP or MeDIP (5mC). Error bars represent standard deviation between three technical replicates.

Figure S7. Zfp57 sites demarcate the protected ICR regions in blastocyst. The extent of methylation at two paternally methylated ICRs (top – Igf2-H19 ICR; bottom – IG-DMR at Dlk1-Gtl2) in sperm and blastocyst as reported in [34] (orange arrows) and [35] (red arrows). Vertical bars denote CpGs and red ticks denote Zfp57 binding motifs.

Figure S8. Imprinting of Zdbf2linc in placenta. (A) Genomic map showing the location and features associated with the novel maternally methylated Gpr1 DMR in ES cells. Arrangement is same as for Fig. 2. Red dotted box highlights the position of the novel Gpr1 DMR and Zdbf2linc RNA putative promoter. (B) Quantitative bisulphite-pyrosequencing analysis of the Gpr1 DMR methylation in germ cells, somatic tissues and placenta. (C) qRT-PCR analysis of Zdbf2linc expression in reciprocal hybrid E16.5 embryo and placenta tissues and (D) Representative SNP-pyrosequencing traces demonstrating its paternal-allele specific expression in placenta. Sequence analysed: [**T**/**C**]GTCTG.

Figure S9. Fkbp6 imprinting and DMR analysis. (A) Left panel: Standard curve showing serial dilution of BC and CB adult testis cDNA. Placenta qPCR Cp-values are marked to demonstrate that they are still within linear range of PCR amplification. Right panel: SNP-pyrosequencing analysis of each dilution point: no random PCR bias even at the lowest dilution, whilst clear paternal allele enrichment in BC & CB Placenta. (B) Bisulphite pyrosequencing summary for adult frontal cortex, testis and ES cells. Annotations as per Fig. 3C.

Figure S10. Allele-specificity of gene expression around strain-specific ZFP57 peaks. (A) Each graph shows relative allele contribution towards expression for each gene adjacent to non-imprinted monoallelic ZFP57 binding site (see also Table 3). Allele ratios are shown as percentages of BL6 allele (0 % = monoallelic Cast, 100 % = monoallelic BL6, 50 % = biallelic expression). Allele ratios found in cDNA from four hybrid ES and NS cells are compared to their genomic counterparts (gDNA, expected 50 % allele ratio) and those having statistically significant differences are indicated (* $P < 0.05$, ** $P < 0.01$, *** $p < 0.001$, ns – not significant; repeated measures anova with Bonferroni post-test, also see Methods). (B) Relative control gene mRNA expression levels are shown on a log scale normalized relative to 18S rRNA. Expression of Zfp57 and Nestin genes are shown as markers of ES and NS cells respectively; Kcnq1ot1 is shown as an example of imprinted (paternally expressed) imprinted gene. Bottom right graph: SNP-pyrosequencing analysis of Kcnq1ot1 RT-PCR product showing predominantly paternal allele-specific expression (Cast allele in BC and BL6 allele in CB cross). Scale indicates percentage of BL6 allele.

Figure S11. UCSC view of human FKBP6 gene locus. Region of human chromosome 7 (chr7: 72,741,752-72,742,742; hg19) containing the CpG island promoter for FKBP6 and TRIM50 genes. Methylation data tracks shown are from ENCODE/HAIB 450K bead chip array: blue, purple and orange indicate unmethylated, partially methylated and fully methylated CpGs respectively. MeDIP tracks are from UCSF analysis of adult human cortex methylation.

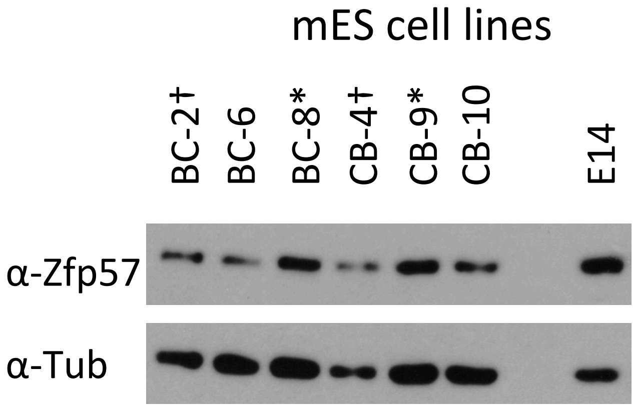

*Lines used in ChIP-seq experiment
†Lines used for independent ChIP-qPCR and SNP-Pyrosequencing

Figure S1

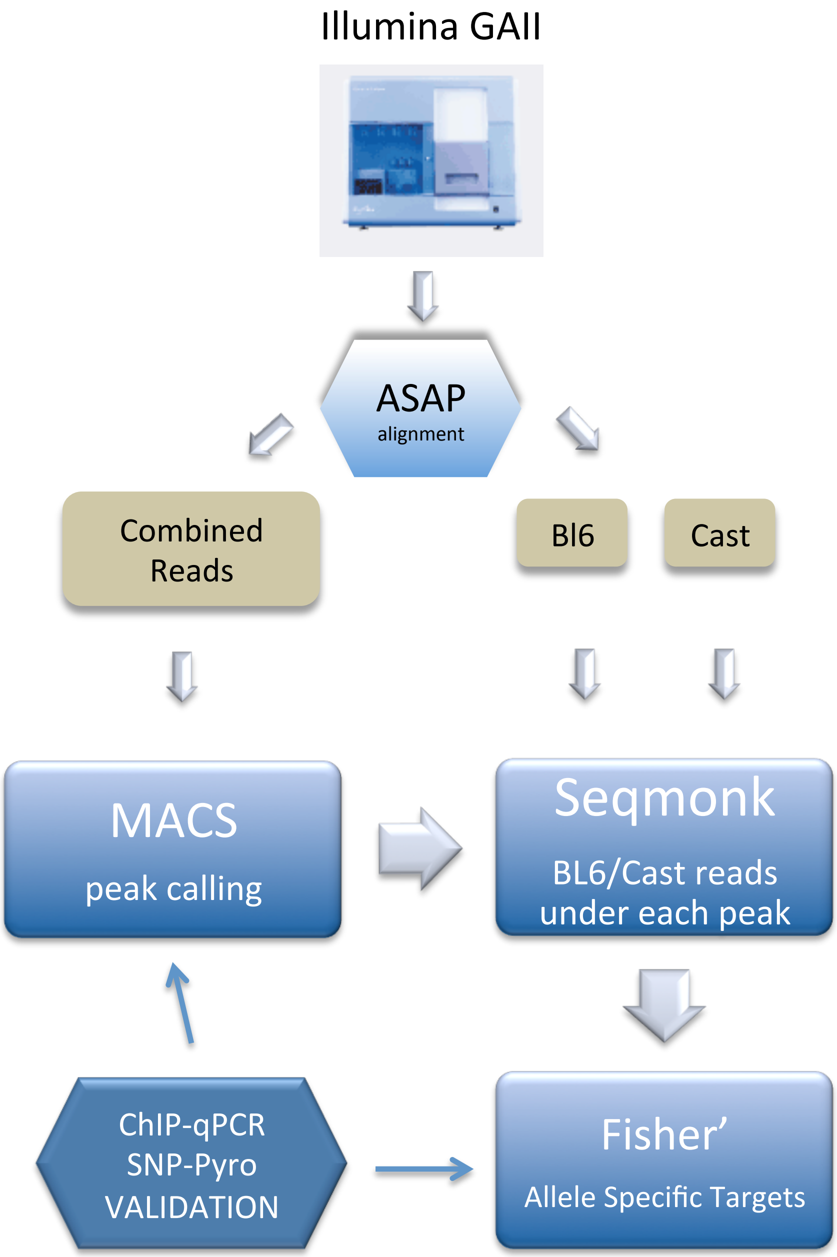
Illumina GAII
ASAP
alignment
Combined Reads
Bl6
Cast
MACS
peak calling
Seqmonk
BL6/Cast reads under each peak
ChIP-qPCR
SNP-Pyro
VALIDATION
Fisher'
Allele Specific Targets

Figure S2

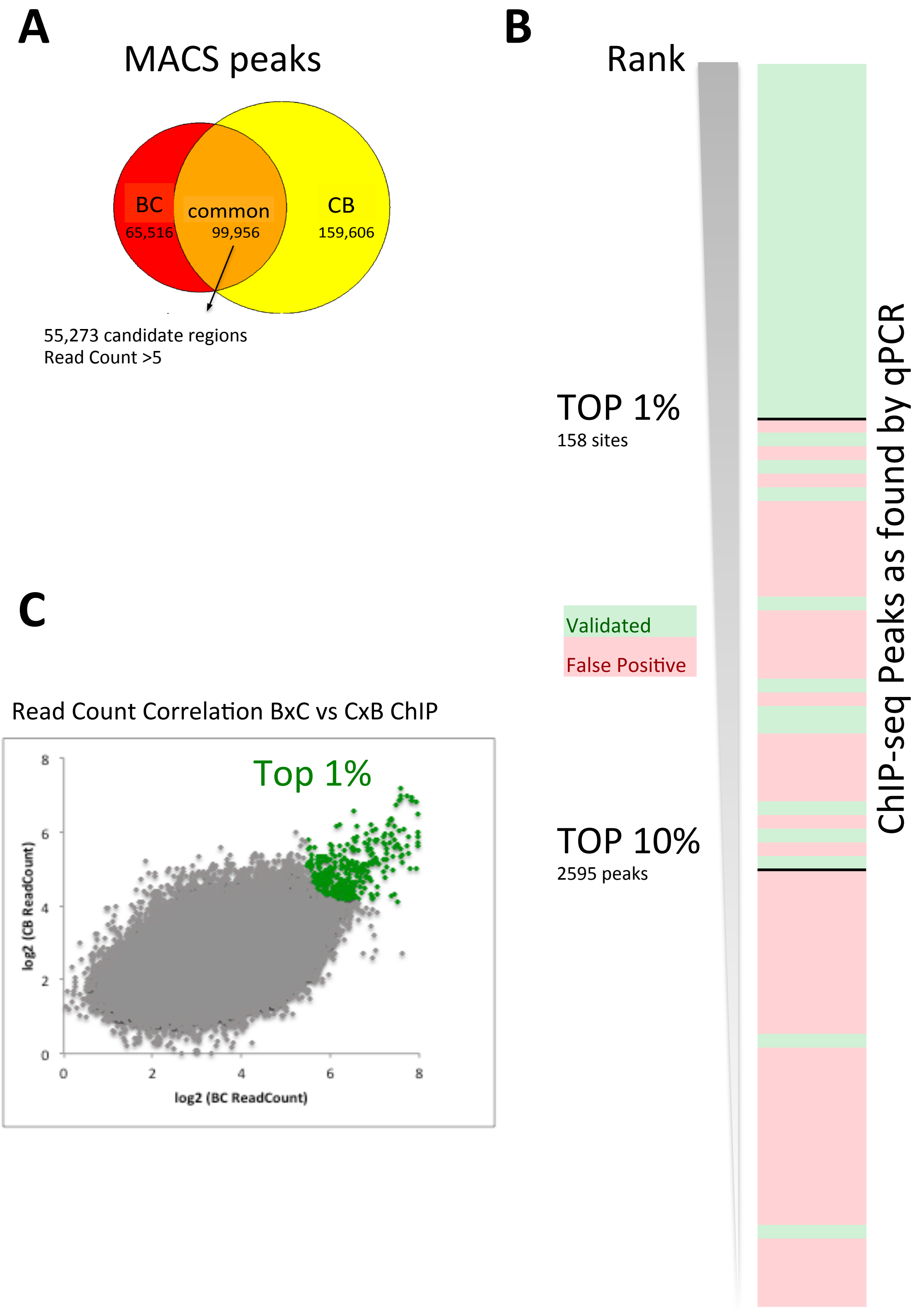
A
MACS peaks
BC
65,516
common
99,956
CB
159,606
55,273 candidate regions
Read Count >5
B
Rank
TOP 1%
158 sites
Validated
False Positive
TOP 10%
2595 peaks
ChIP-seq Peaks as found by qPCR
C
Read Count Correlation BxC vs CxB ChIP
Top 1%
log2 (CB ReadCount)
log2 (BC ReadCount)
0
2
4
6
8

Figure S3

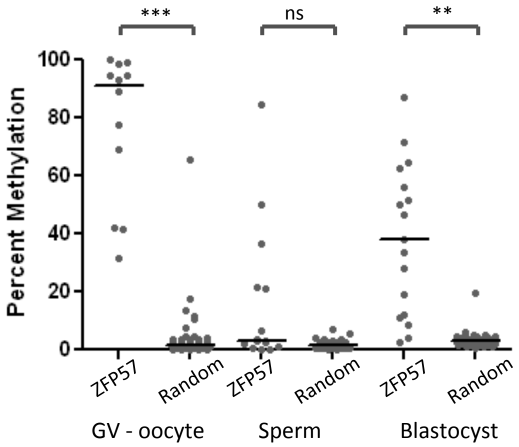
***
ns
**
Percent Methylation
100
80
60
40
20
0
ZFP57
Random
ZFP57
Random
ZFP57
Random
GV - oocyte
Sperm
Blastocyst

Figure S4

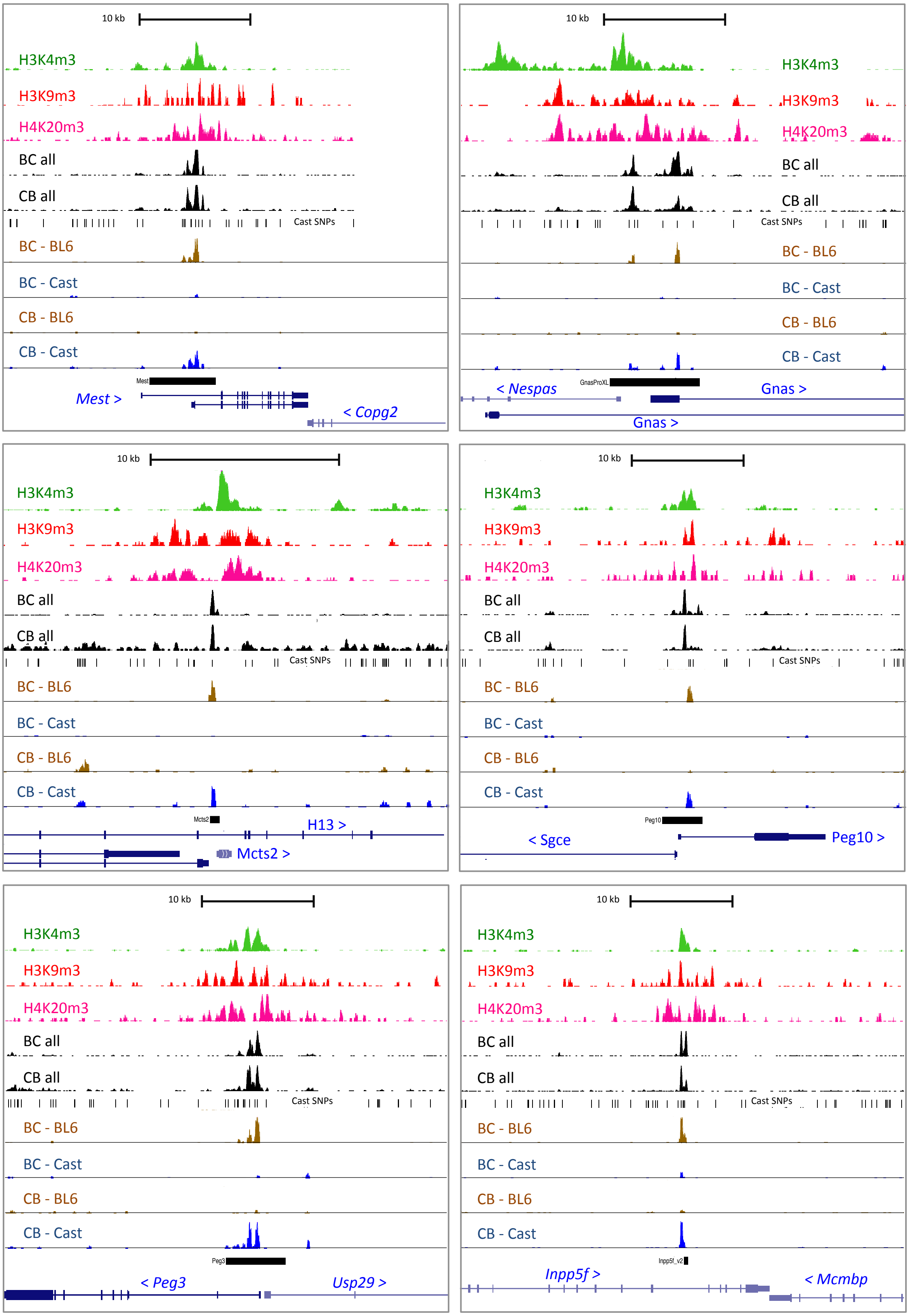
10 kb
H3K4m3
H3K9m3
H4K20m3
BC all
CB all
Cast SNPs
BC - BL6
BC - Cast
CB - BL6
CB - Cast
Mest
Mest >
< Copg2
GnasProXL
< Nespas
Gnas >
Gnas >
Mcts2
H13 >
Mcts2 >
Peg10
< Sgce
Peg10 >
Peg3
< Peg3
Usp29 >
Inpp5f_v2
Inpp5f >
< Mcmbp

Figure S5

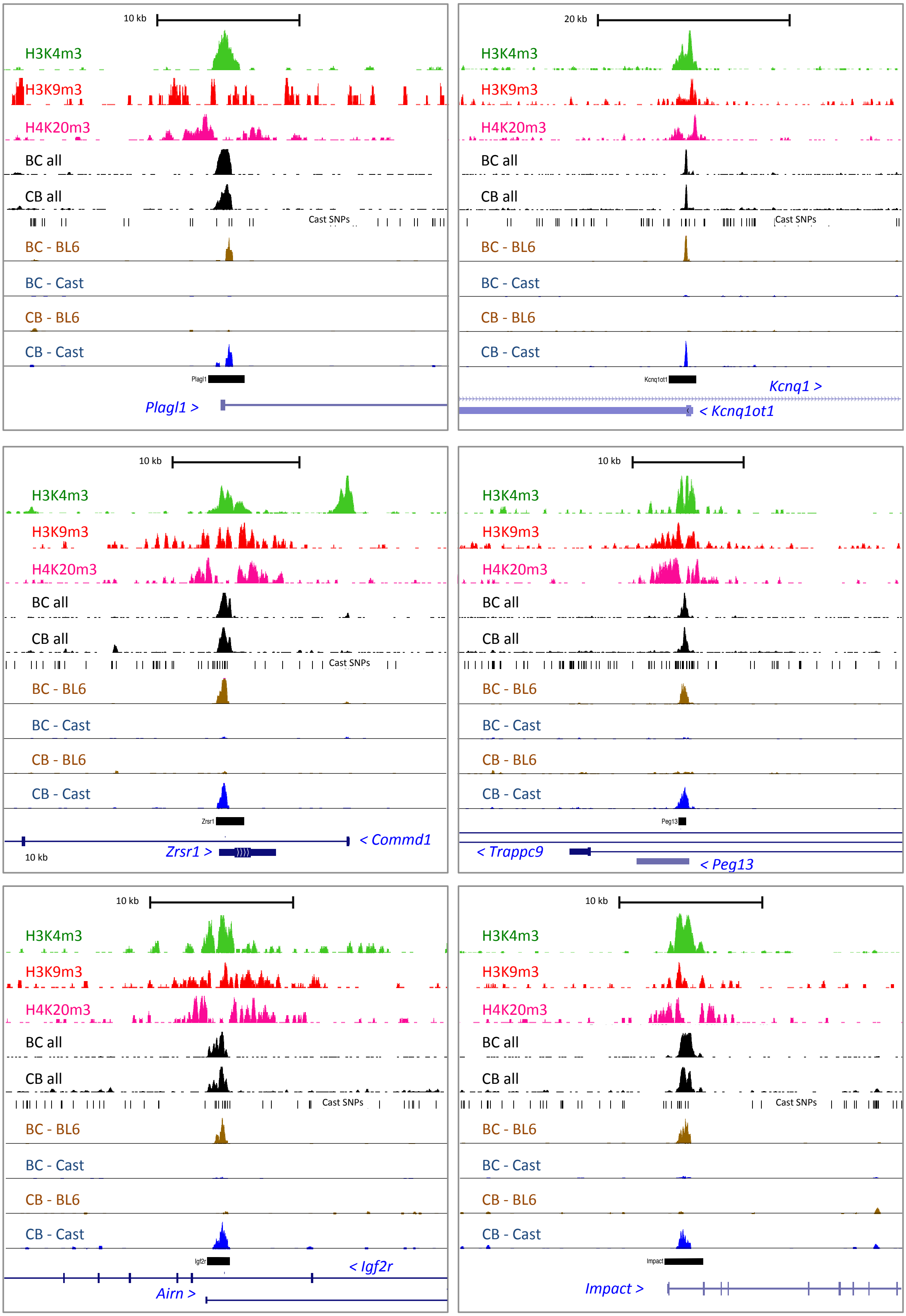

Figure S5 cont.

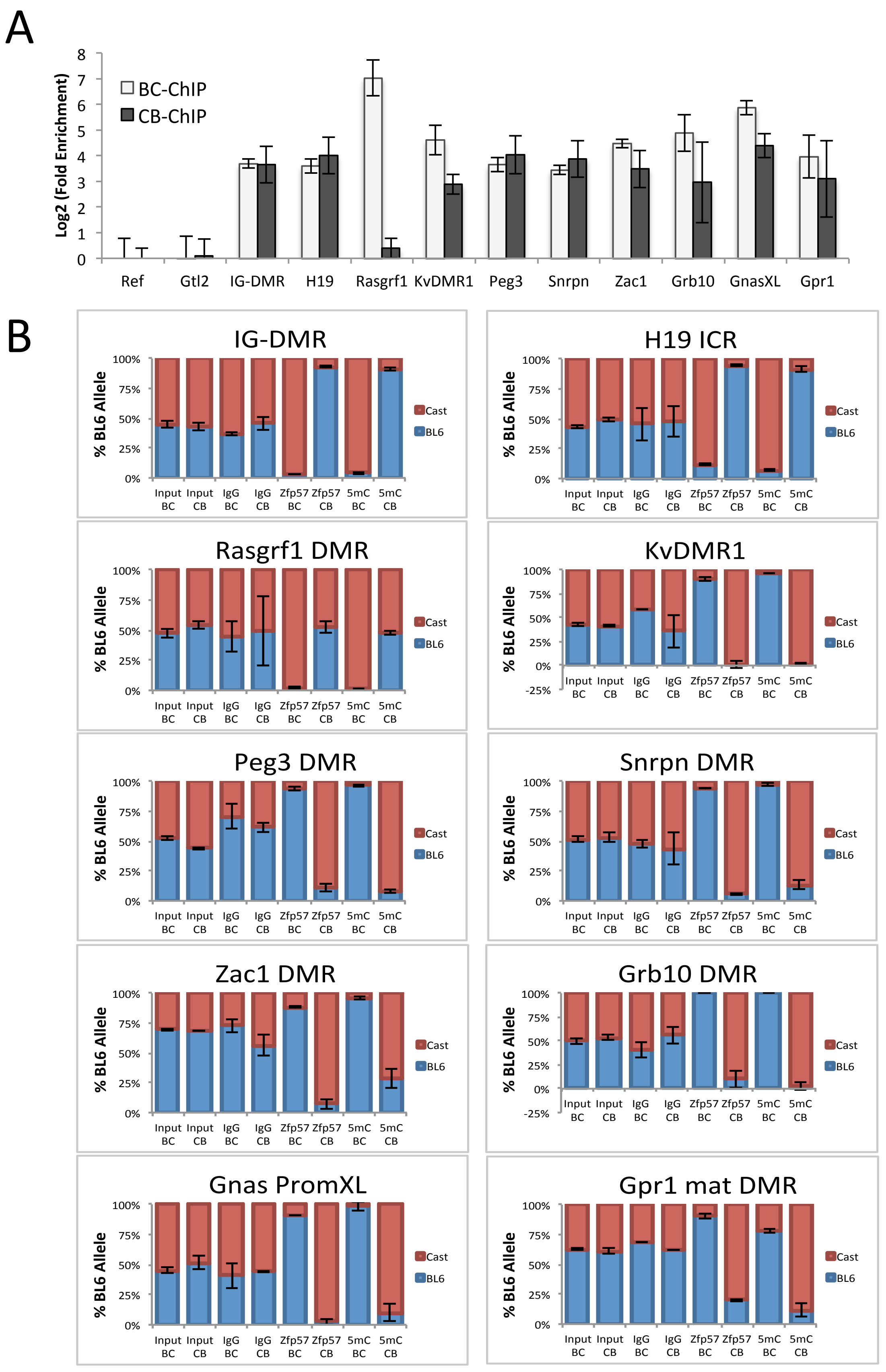
A
Log2 (Fold Enrichment)
BC-ChIP
CB-ChIP
Ref
Gtl2
IG-DMR
H19
Rasgrf1
KvDMR1
Peg3
Snrpn
Zac1
Grb10
GnasXL
Gpr1
B
IG-DMR
H19 ICR
Rasgrf1 DMR
KvDMR1
Peg3 DMR
Snrpn DMR
Zac1 DMR
Grb10 DMR
Gnas PromXL
Gpr1 mat DMR
% BL6 Allele
Cast
BL6
Input BC
Input CB
IgG BC
IgG CB
Zfp57 BC
Zfp57 CB
5mC BC
5mC CB

Figure S6

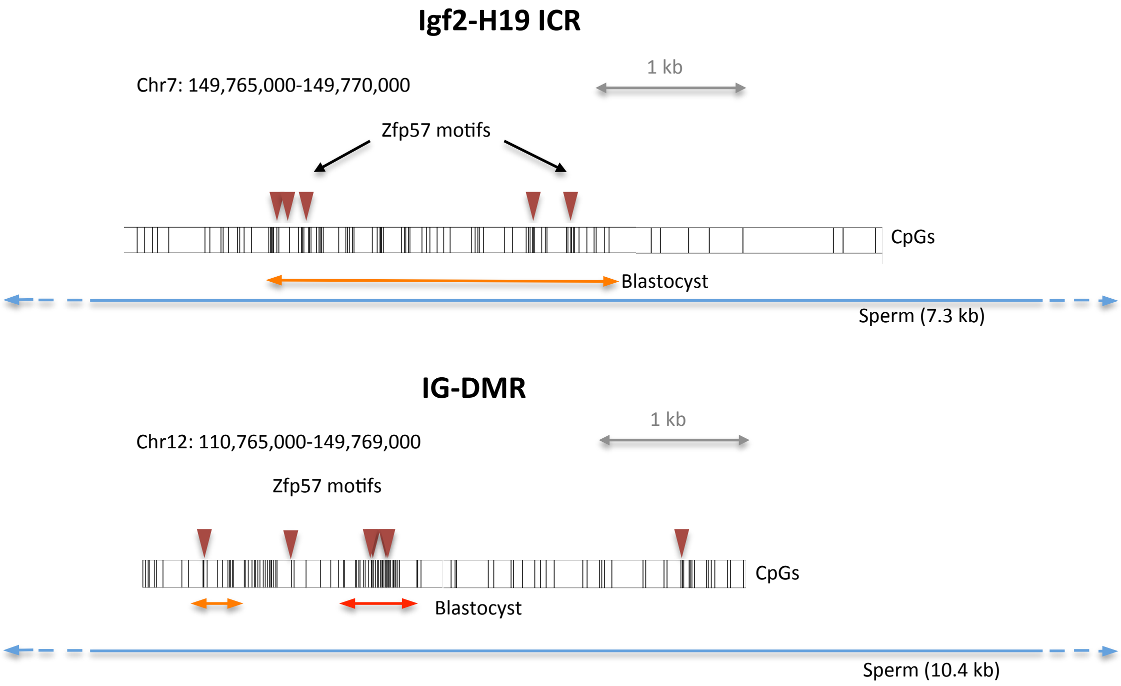
Igf2-H19 ICR
Chr7: 149,765,000-149,770,000
1 kb
Zfp57 motifs
CpGs
Blastocyst
Sperm (7.3 kb)
IG-DMR
Chr12: 110,765,000-149,769,000
1 kb
Zfp57 motifs
CpGs
Blastocyst
Sperm (10.4 kb)

Figure S7

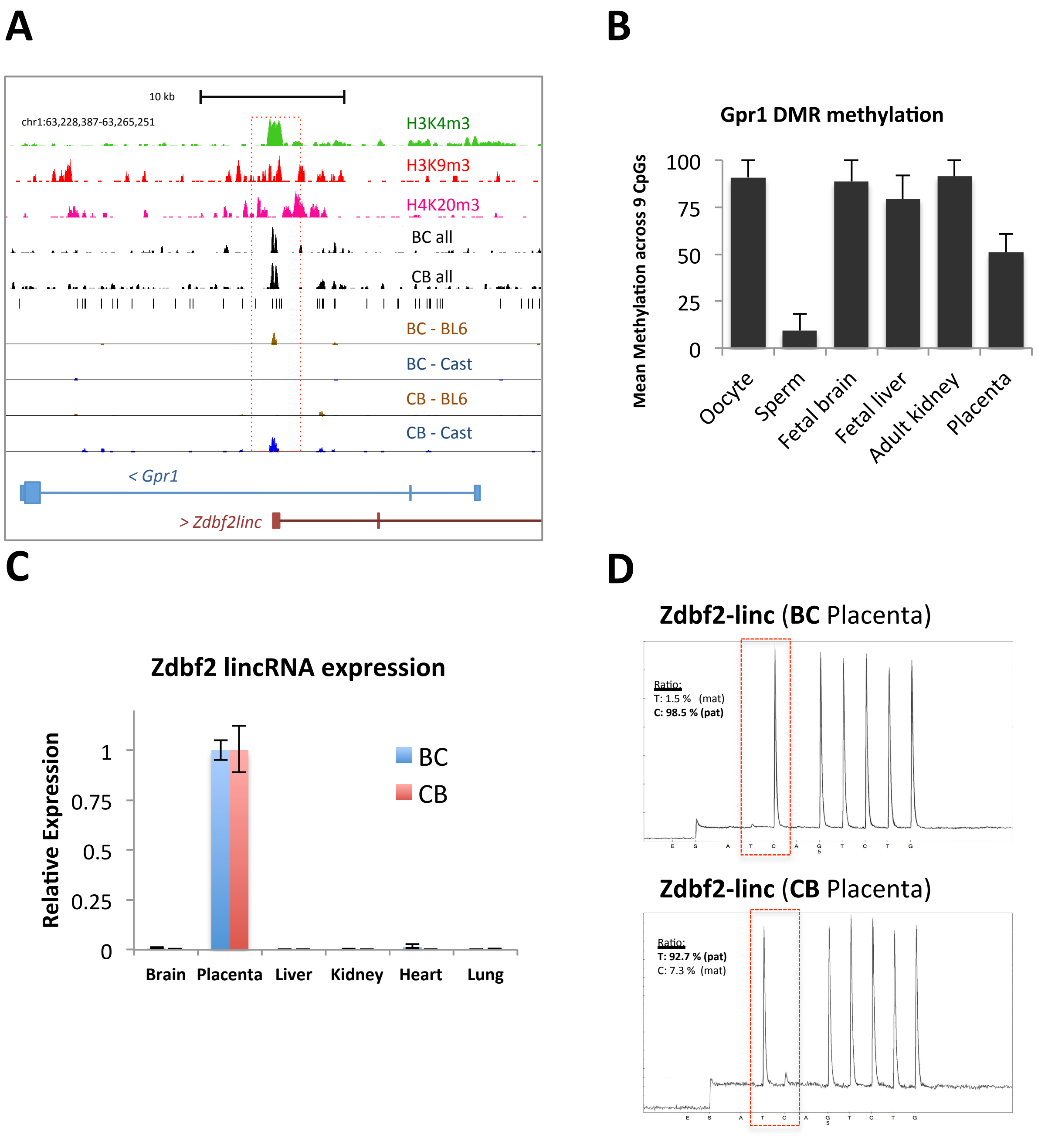
A
10 kb
chr1:63,228,387-63,265,251
H3K4m3
H3K9m3
H4K20m3
BC all
CB all
BC - BL6
BC - Cast
CB - BL6
CB - Cast
< Gpr1
> Zdbf2linc
B
Gpr1 DMR methylation
Mean Methylation across 9 CpGs
100
75
50
25
0
Oocyte
Sperm
Fetal brain
Fetal liver
Adult kidney
Placenta
C
Zdbf2 lincRNA expression
Relative Expression
1
0.75
0.5
0.25
0
BC
CB
Brain
Placenta
Liver
Kidney
Heart
Lung
D
Zdbf2-linc (BC Placenta)
Ratio:
T: 1.5 % (mat)
C: 98.5 % (pat)
Zdbf2-linc (CB Placenta)
Ratio:
T: 92.7 % (pat)
C: 7.3 % (mat)

Figure S8

A

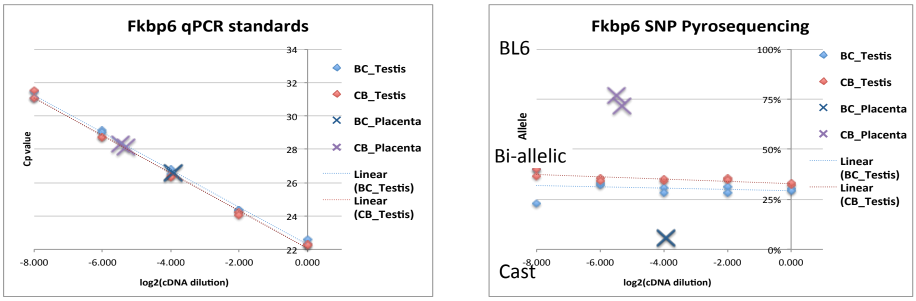
Fkbp6 qPCR standards
34
32
30
28
26
24
22
Cp value
-8.000
-6.000
-4.000
-2.000
0.000
log2(cDNA dilution)
BC_Testis
CB_Testis
BC_Placenta
CB_Placenta
Linear (BC_Testis)
Linear (CB_Testis)
Fkbp6 SNP Pyrosequencing
BL6
Bi-allelic
Cast
Allele
100%
75%
50%
25%
0%
-8.000
-6.000
-4.000
-2.000
0.000
log2(cDNA dilution)
BC_Testis
CB_Testis
BC_Placenta
CB_Placenta
Linear (BC_Testis)
Linear (CB_Testis)

B

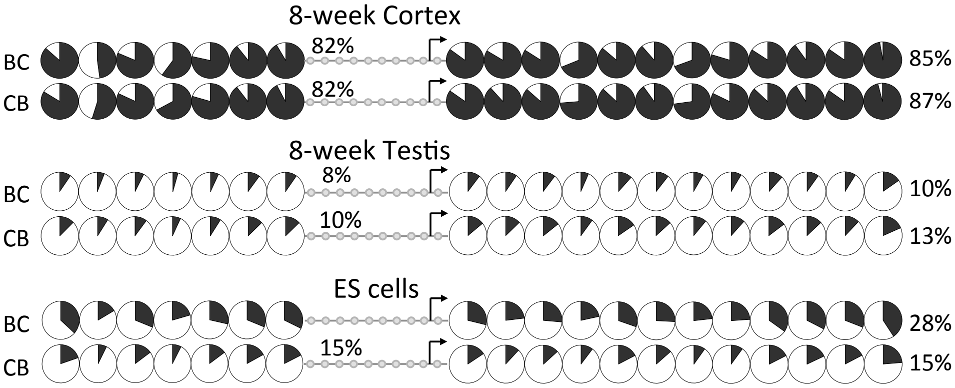
8-week Cortex
BC
82%
85%
CB
82%
87%
8-week Testis
BC
8%
10%
CB
10%
13%
ES cells
BC
28%
CB
15%
15%

Figure S9

A

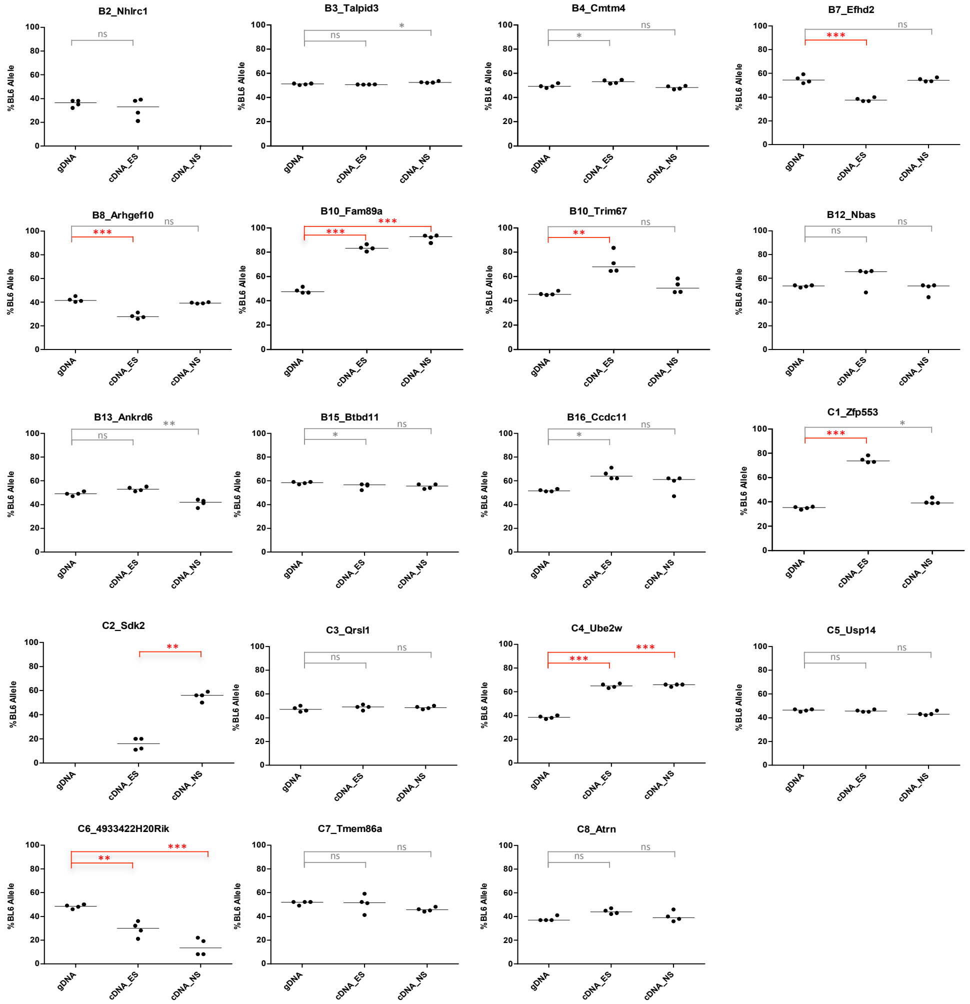

B

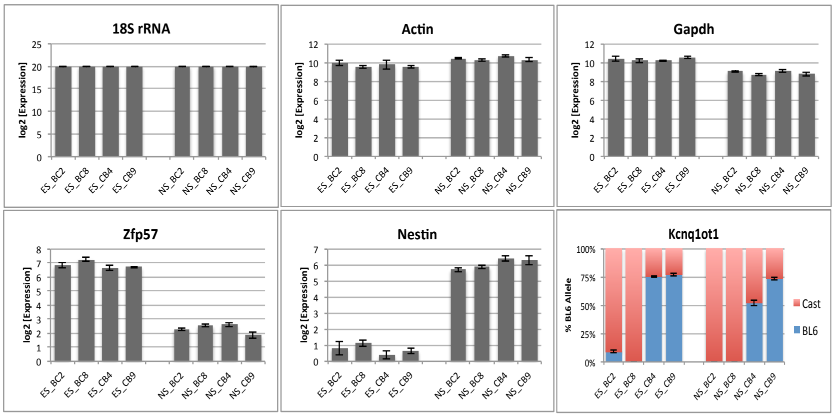

Figure S10

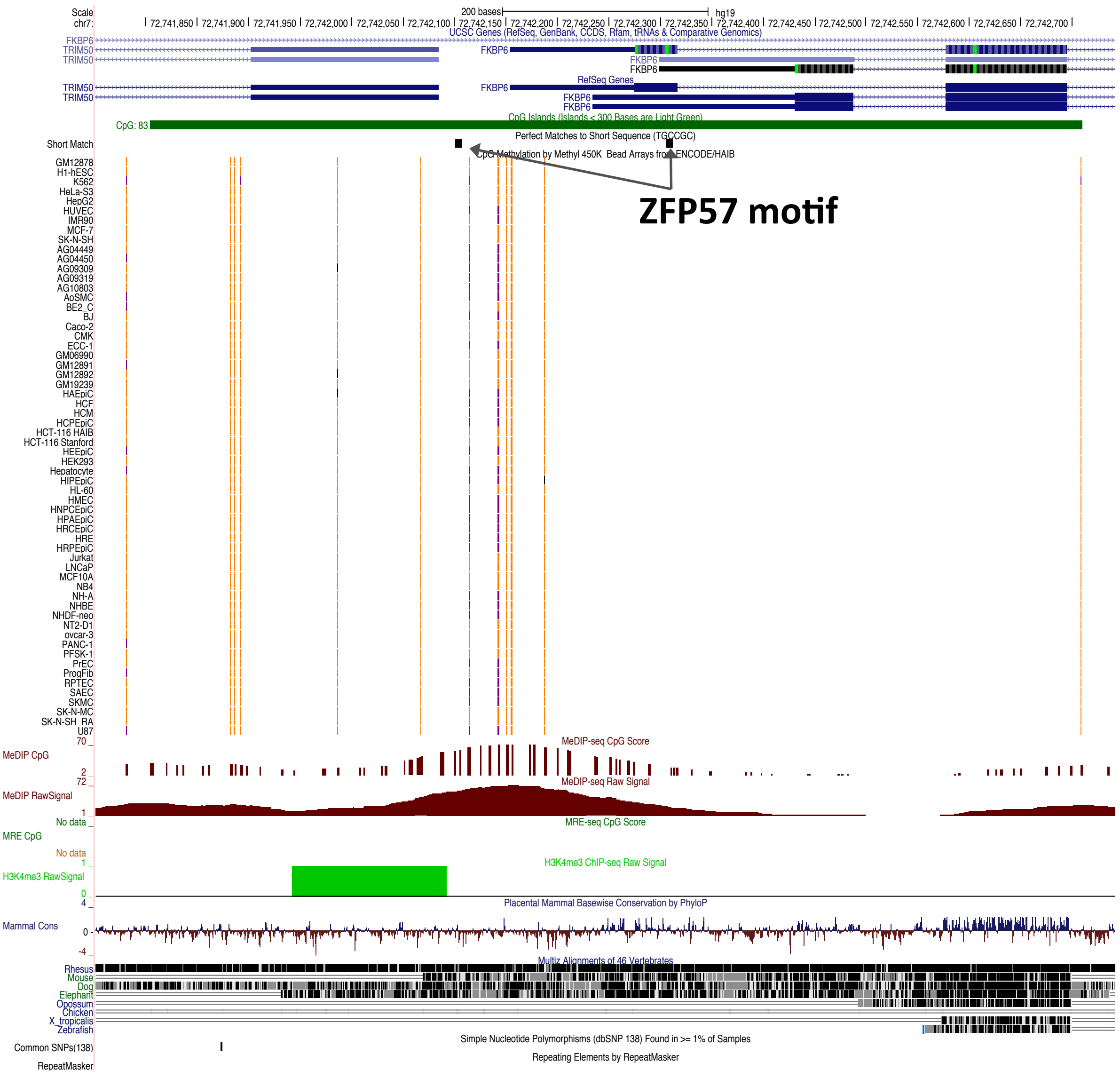
Scale
chr7:
200 bases
hg19
72,741,850
72,741,900
72,741,950
72,742,000
72,742,050
72,742,100
72,742,150
72,742,200
72,742,250
72,742,300
72,742,350
72,742,400
72,742,450
72,742,500
72,742,550
72,742,600
72,742,650
72,742,700
UCSC Genes (RefSeq, GenBank, CCDS, Rfam, tRNAs & Comparative Genomics)
FKBP6
TRIM50
RefSeq Genes
CpG Islands (Islands < 300 Bases are Light Green)
CpG: 83
Short Match
Perfect Matches to Short Sequence (TGCCGC)
CpG Methylation by Methyl 450K Bead Arrays from ENCODE/HAIB
ZFP57 motif
GM12878
H1-hESC
K562
HeLa-S3
HepG2
HUVEC
IMR90
MCF-7
SK-N-SH
AG04449
AG04450
AG09309
AG09319
AG10803
AoSMC
BE2_C
BJ
Caco-2
CMK
ECC-1
GM06990
GM12891
GM12892
GM19239
HAEpiC
HCF
HCM
HCPEpiC
HCT-116 HAIB
HCT-116 Stanford
HEEpiC
HEK293
Hepatocyte
HIPEpiC
HL-60
HMEC
HNPCEpiC
HPAEpiC
HRCEpiC
HRE
HRPEpiC
Jurkat
LNCaP
MCF10A
NB4
NH-A
NHBE
NHDF-neo
NT2-D1
ovcar-3
PANC-1
PFSK-1
PrEC
ProgFib
RPTEC
SAEC
SKMC
SK-N-MC
SK-N-SH_RA
U87
MeDIP CpG
MeDIP-seq CpG Score
MeDIP RawSignal
MeDIP-seq Raw Signal
MRE CpG
MRE-seq CpG Score
No data
H3K4me3 RawSignal
H3K4me3 ChIP-seq Raw Signal
Mammal Cons
Placental Mammal Basewise Conservation by PhyloP
Multiz Alignments of 46 Vertebrates
Rhesus
Mouse
Dog
Elephant
Opossum
Chicken
X_tropicalis
Zebrafish
Common SNPs(138)
Simple Nucleotide Polymorphisms (dbSNP 138) Found in >= 1% of Samples
RepeatMasker
Repeating Elements by RepeatMasker

Figure S11
