# Supplementary material for: Allele-specific binding of ZFP57 in the epigenetic regulation of imprinted and non-imprinted monoallelic expression
Source: Genome Biol. 2015 May 30;16(1):112. doi: 10.1186/s13059-015-0672-7 (PMC4491874; doi:10.1186/s13059-015-0672-7)
Supplement: Additional file 3: — Genomic sequence alignments of C57BL/6 and Cast/EiJ DNA around monoallleic Zfp57 binding sites. [file 13059_2015_672_MOESM3_ESM.docx]

Key:

**Red Bold**: SNP and indel positions

Yellow highlight: TGCCGCR motif

Grey highlight: TGCCGCY motif

Genome assembly coordinates: NCBI37/mm9

Relates to Table S1, strain-specific_sites

**Peak26791 (B1) Some motifs disrupted**

>C57BL/6 (chr14:68739513-68739886)

GCTTAATGTAGTGGGCACTTTTGAATAAATAGAACCACGTAAGGAAATACTCTTTCTACCAGGGCAGTGAGTGCTTACTGCCATTTCATCAACATGTTTATCATATCCTGGGGCAGATTCTCCAAGAGTGCCAGACAGTCCTCTGCTG**T**AG**T**GCCTAGCCACTCCCAGTAGACAACTA**A**CCACACCCAGTCAGTAAATGCA**T**CTC**TGCGGCA**GGCATGCAGTTG**CGCGGCA**GGCATGCAGTTG**CGCGGCA**GGCATGCAGTTG**CGCGGCA**GGCATGCAGTTGCTGTAGGTGTAGTATGAATGTGCTAAAATGGTCTCTAGTGGCCAACCCACTTTGTCCCCCAAACGGTGTCCTCCCTCATCATTTGAATTGCCA

>Cast/EiJ

GCTTAATGTAGTGGGCACTTTTGAATAAATAGAACCACGTAAGGAAATACTCTTTCTACCAGGGCAGTGAGTGCTTACTGCCATTTCATCAACATGTTTATCATATCCTGGGGCAGATTCTCCAAGAGTGCCAGACAGTCCTCTGCTG**C**AG**C**GCCTAGCCACTCCCAGTAGACAACTA**G**CCACACCCAGTCAGTAAATGCA**G**CTC**TGCGGCA**GGCATGCAGTTG**.......**............**.......**.............**.......**...........GTGTAGGTGTAGTATGAATGTGCTAAAATGGTCTCTAGTGGCCAACCCACTTTGTCCCCCAAACGGTGTCCTCCCTCATCATTTGAATTGCCA

**Peak22646 (B2) All motifs intact**

>C57BL/6 (chr13:47106142-47106589)

AAGGCCCCTCCCCTACT**T**GCTTCTATGATGATGGCCACGAATCTGGGGTTGATGGCGCAAAAGGAACTG**T**CCCAGGTGACT**C**G**G**GAGACGCGGATGTCATCATAGCACTGATCATTCTTCACCGCCTGTCCAAAGAC**A**TGCCGGAACTTGCTC**TGCCGCA**CCACTCGCCGCATC**G**TGTCAGAGCCCGGGTGCCTGAGGCGCC**A**CCGAAGTCCCTGCTGCAAAGCCCCG**A**GAAGCCTCCATCC**ATCC**G**CTGCCGCC**TCCCGCCTGGCACTGAG**TGCCGCG**CGGGGGAGGGG**CGCGGCAA**CC**G**CTGCC**C**AGTCC**T**TCGCGCA**A**GCGTGC**A**GGCCCTGCCCCCCTCAAATCTTTGAATTGGGTCAACAGACACTAACATTATCCAAATGTAATGGTTCTGAAATAAAACACTGTAAGATCAATCCACAGTTGCTATCTAAGTTTTGCTGTT

>Cast/EiJ

AAGGCCCCTCCCCTACT**C**GCTTCTATGATGATGGCCACGAATCTGGGGTTGATGGCGCAAAAGGAACTG**C**CCCAGGTGACT**T**G**A**GAGACGCGGATGTCATCATAGCACTGATCATTCTTCACCGCCTGTCCAAAGAC**G**TGCCGGAACTTGCTC**TGCCGCA**CCACTCGCCGCATC**A**TGTCAGAGCCCGGGTGCCTGAGGCGCC**G**CCGAAGTCCCTGCTGCAAAGCCCCG**C**GAAGCCTCCATCC....G**ATGCCGCC**TCCCGCCTGGCACTGAG**TGCCGCG**CGGGGGAGGGG**CGCGGCAG**CC**A**CTGCC**T**AGTCC**C**TCGCGCA**C**GCGTGC**G**GGCCCTGCCCCCCTCAAATCTTTGAATTGGGTCAACAGACACTAACATTATCCAAATGTAATGGTTCTGAAATAAAACACTGTAAGATCAATCCACAGTTGCTATCTAAGTTTTGCTGTT

**Peak19026 (B3) All motifs disrupted**

>C57BL/6 (chr12:72343084-72343492)

ACGAACTTGCGCTTCAGTTTGGAATGCTTTCATTTGTTTCCTAA**T**CAGGCCACTTCAGTTCTCCATCTTCCTCCTTCGAC**G**CTCAGGGCTGGGGTTAGAGGCCAGCGCCACGATGCTGCC**GCGCCACGATGCTGCCGCGCCACGATGCTGCCGCGCCACGATGCTACC**TTC**G**GATGTTTCCTCCTCTCCTA.GGCTTCTTTTTAGAA**G**TGATTCTGTAATCAGTAGCAC**A**CCCCCACTGCGGCTGCTGCAGTGGCTTGCAGTCTCTGATGCAGTGAGAGCGACATTAGGGTCTTTCATG**G**AGACAGTGGCTCTGTCT..GAGCACTCACTGCACTGGGGTTTTGGGGTTTGAATGATGTTGACAGTGCTGAGCTCTG**CTCTG**AGGTCGGGTTCCATGTGCTCTGCAAGAAAT

>Cast/EiJ

ACGAACTTGCGCTTCAGTTTGGAATGCTTTCATTTGTTTCCTAA**A**CAGGCCACTTCAGTTCTCCATCTTCCTCCTTCGAC**T**CTCAGGGCTGGGGTTAGAGGCCAGCGCCACGATGC**TGCC...**..............................................TTC**A**GATGTTTCCTCCTCTCCTA**C**GGCTTCTTTTTAGAA**A**TGATTCTGTAATCAGTAGCAC**G**CCCCCACTGCGGCTGCTGCAGTGGCTTGCAGTCTCTGATGCAGTGAGAGCGACATTAGGGTCTTTCATG**A**AGACAGTGGCTCTGTCT**CT**GAGCACTCACTGCACTGGGGTTTTGGGGTTTGAATGATGTTGACAGTGCTGAGCTCTG.....AGGTCGGGTTCCATGTGCTCTGCAAGAAAT

**Peak91151 (B4) All motifs disrupted**

>C57BL/6 (chr8:106889256-106889799)

ATTACTCCCTTTGGAAGCTGGTTTACACAGAGACCCTGGCTGCTGTGCTCCCTGGGCAGCGCACTCTGGTTTCCAGCTGTGAAGGATGCCCCAACTCACACTGCGGCCACACAGCTCTGGGGTTCTCAGGCTAAGCCCAAGGCCATGTCCACATCCCTGGTCTTGG**T**ATGCTCACAGGGCACCAGTGTCTTCCTGGTTCTTAAATGTGCTGTCAAGCCTGCTTTCCTCTGCTTGATACAGGGGCTCTGTATCTTGAACACAGCCCCACCCCTGCTTCCCAGCACACGTAATAAAACTACATAGAGATGCACAAAGGGAAAGCAGCGTCCTGGGCTGGTCAGCCCCCAGCCTGGTCAGGCCGCGCACACCTTACAGAGAGAAG**CGCGGCA**ACCCACTGACCCAGCAGGGCAGCAATGGCAAAAAGGCTGCCAGGT**C**CCGAGTGCACACACACATGCGCAGCAGTGCCTCCATGGCCTTCGGAAGCAAGTGGAATTAATTATAACCTGCTCAAAGTTAAAATATCACCAGTCCAGACAGTGTGCAGAAT

>Cast/EiJ

ATTACTCCCTTTGGAAGCTGGTTTACACAGAGACCCTGGCTGCTGTGCTCCCTGGGCAGCGCACTCTGGTTTCCAGCTGTGAAGGATGCCCCAACTCACACTGCGGCCACACAGCTCTGGGGTTCTCAGGCTAAGCCCAAGGCCATGTCCACATCCCTGGTCTTGG**A**ATGCTCACAGGGCACCAGTGTCTTCCTGGTTCTTAAATGTGCTGTCAAGCCTGCTTTCCTCTGCTTGATACAGGGGCTCTGTATCTTGAACACAGCCCCACCCCTGCTTCCCAGCACACGTAATAAAACTACATAGAGATGCACAAAGGGAAAGCAGCGTCCTGTGCTGGTCAGCCCCCAGCCTGGTCAGGCCGCGCACACCTTACAGAGAGAAG**CACGGCA**ACCCACTGACCCAGCAGGGCAGCAATGGCAAAAAGGCTGCCAGGG**T**CCGAGTGCACACACACATGCGCAGCAGTGCCTCCATGGCCTTCGGAAGCAAGTGGAATTAATTATAACCTGCTCAAAGTTAAAATATCACCAGTCCAGACAGTGTGCAGAAT

**Peak3528 (B5) All motifs intact**

>C57BL/6 (chr1:157376729-157377155)

TGCTCCAGATGGGACAGACTCTAAGGTGTTCCAAATATTTCTGTGCCCCGGGCTGCAACGACTCTCCCGGAGGGGCAGCCCAAACA**C**T**C**ACCG**..**AGTAACTCAC**C**GAGTCGCGGCCCAGCCAC**TGCGGCA**AGTGCAGCTACT**G**CCTCAGATTTGGCTCCGTTTTAGGGCAGTGCC**C**TTACACACAGGCTTCCTATGCCGGAGTCCAGA**C**CCCTGTTCTTCTCAGGCCCAGATCACGTCAGGTGGCAACACTGGGTCTCCACACTGGGTTGTGACCCCTTCTGCTGTCACCTCGGGCTATTCCGCCGTACCCCAGTTCCATACCGTACACAAGAGATTCCCTCTACATTCTGCCAGTGGCAAGATTTGAACTACTGCCTCTCATCTTTTCCCGCCTCTTACAGAAGCCCTTCAAGGGCAGAAACCTGTCTA

>Cast/EiJ

TGCTCCAGATGGGACAGACTCTAAGGTGTTCCAAATATTTCTGTGCCCCGGGCTGCAACGACTCTCCCGGAGGGGCAGCCCAAACA**G**T**T**ACCG**GA**AGTAACTCAC**T**GAGTTGCGGCCCAGCCAC**TGCGGCA**AGTGCAGCTACT.CCTCAGATTTGGCTCCGTTTTAGGGCAGTGCC**T**TTACACACAGGCTTCCTATGCCGGAGTCCAGA**T**CCCTGTTCTTCTCAGGCCCAGATCACGTCAGGTGGCAACACTGGGTCTCCACACTGGGTTGTGACCCCTTCTGCTGTCACCTCGGGCTATTCCGCCGTACCCCAGTTCCATACCGTACACAAGAGATTCCCTCTACATTCTGCCAGTGGCAAGATTTGAACTACTGCCTCTCATCTTTTCCCGCCTCTTACAGAAGCCCTTCAAGGGCAGAAACCTGTCTAAT

**Peak24804 (B6) Some motifs disrupted**

>C57BL/6 (chr14:8633488-8633934)

TTAGAAATTGGCCAGGAATGTGG**G**GCAGGAACTAGCAGGAGAAGGGAAGACTGCAGAAGGGGATTTGCTCGCCAGACAATT.ATGCCATAGAACGTCTCCTCACGAAGCAAAGCACTGTGCGTAATAAATGTACACAAGGAAGCATAAAATTTAATTTTTAAAACAAGCACCGGACCTTGTCAGTGCTGTTGCTGAGTGAAGTGCT**TGCCGCG**GAGCTGCCCTTCAGACCT**TTGCCGCC**ATGGCCTGCAGACCTAGAAGCCCACC**T**GGTTATGGGAGTCGCCGCGACGGTGGTGTGAGCCCGCGGTCCCCTGCGAGATGGAGTCTCGGA**T**GGAAGCGCAGAGC**A**GACAGA**G**GGGAC**CA**GAAGCCCGAGGACTCCGAGGAAGGCGAGCTCCAGACT**T**CGGACCAC**G**GACCTGAAAGCTTTACAACTCCTGAAGGTCATAAGCCCCGTTCTAA

>Cast/EiJ

TTAGAAATTGGCCAGGAATGTGG**A**GCAGGAACTAGCAGGAGAAGGGAAGACTGCAGAAGGGGATTTGCTCGCCAGACAATT**T**ATGCCATAGAACGTCTCCTCACGAAGCAAAGCACTGTGCGTAATAAATGTACACAAGGAAGCATAAAATTTAATTTTTAAAACAAGCACCGGACCTTGTCAGTGCTGTTGCCGAGTGAAGTGCT**TGCCGCC**GAGCTGCCCTTCAGACCT**CTGCCGCC**ATGGCCTGCAGACCTAGAAGCCCACC**C**GGTTATGGGAGTCGCCGCGACGGTGGTGTGAGCCCGCGGTCCCCTGCGAGATGGAGTCTCGGA**C**GGAAGCGCAGAGC**G**GACAGA**A**GGGAC**.G**GAAGCCCGAGGACTCCGAGGAAGGTGAGCTCCAGACT**G**CGGACCAC**C**GACCTGAAAGCTTTACAACTCCTGAAGGTCATAAGCCCCGTTCTAA

**Peak64416 (B7) All motifs disrupted**

>C57BL/6 (chr4:141410169-141410450)

GAAGCGAGGCTGTAAAATGC**T**AACCC**T**TTAG**C**CCCCAGAAAAAGCT**A**GCCAGGC**T**CTA**T**CTGGGAAATTAG**G**GGCCCTC**C**TGCTGAC**TG**ACC**TTAA**CAATCAATAT**TGCGTTA**TCTGT**GC**AAGA**A**TT**C**TAG**CA**AACTGGTAAATGGAAA**CGT**GGAACTGAGTGG**T**TGGTA**C**GGGCCC**A**GCAGAAGCTAG**CC**CCAC**AGGGT**GG**A**CCAGGGC**A**GCACAG**C**TCA**TG**TGGTAGAAGT**C**TGT**G**A**G**TCT**T**G...............CAG**CGCGGCA**GT**A**GTGGAAA**T**CCATGTGG**TGTAG.......**CAG

>Cast/EiJ

GAAGCGAGGCTGTAAAATGC**C**AACCC**C**TTAG**T**CCCCAGAAAAAGCT**G**GCCAGGC**C**CTA**C**CTGGGAAATTAG**A**GGCCCTC**T**TGCTGAC**CA**ACC**CTTC**CAATCAATAT**GCTCATG**TCTGT**AT**AAGA**T**TT**G**TAG**TG**AACTGGTAAATGGAAA**TGC**GGAACTGAGTGG**G**TGGTA**T**GGGCCC**T**GCAGAAGCTAG**TT**CCAC**TGAAA**GG**G**CCAGGGT**G**GCACAG**G**TCA**CA**TGGTAGAAGT**T**TGT**A**A**A**TCT**A**G**TAACAGTGGAGGTAG**CAGCG**ATGGCCTGCCA**CAGT**G**GTGGAAA**G**CCATGTGG**GGTGGATGATGG**CAG

**Peak88171 (B8) Some motifs disrupted**

>C57BL/6 (chr8:14958674-14959111)

CCCTGAAGGGTCTCTCCTTTCCTCTCCTTCATCTGCCCGCCCCTGCCCCCTCCCCTCCTCCCAGGGGACAGCAGGCCACAATCACCAGCAGCCACAGAGCCTATGGAGACCAAAGAAGTGTGGAACTGGACTGAAGGAGGGTCGTGGGCTCCCTTGTTGGTTCTGGGAAGCAAACGGGGTCCTCGTAATCAGCCCTGTCAAGTGT**TGCCGCT**CTTCTTGACTGCACG**TGCCGCG**TTCACATTGTTATCATCGGTCCGTGCTGCCTGCAGCACTTACCAGGAGCTTCCTTCTCTGGCCTTTGTAAGGTTTCAGTGACCGAGGCCCGTTCTTCAGGCTCCCTCTCTCTAGTTCAGTGTCTCCCCTTGGTCCCCAGCGATTTCCCGACACT**TGCGGCA**CCATGCTTTGTCCCTTTCCTCCGTGTAGTTGGTCTCTGGGGTG

>Cast/EiJ

CCCTGAAGGGTCTCTCCTTTCCTCTCCTTCATCTGCCCGCCCCTGCCCCCTCCCCTCCTCCCAGGGGACAGCAGGCCACAATCACCAGCAGCCACAGAGCCTATGGAGACCAAAGAAGTGTGGAACTGGACTGAAGGAGGGTCGTGGGCTCCCTTGTTGGTTCTGGGAAGCAAACGGGGTCCTCGTAATCAGCCCTGTCAAGTGT**TGCCGCT**CTTCTTGACTGCACG**TGCCGCG**TTCACATTGTTATCATCGGTCCGTGCTGCCTGCAGCACTTACCAGGAGCTTCCTTCTCTGGCCTTTGTAAGGTTTCAGTGACCGAGGCCCGTTCTTCAGGCTCCCTCTCTCTAGTTCAGTGTCTCCCCTTGGTCCCCAGCGATTTCCCGACACT**TGCAGCA**CCATGCTTTGTCCCTTTCCTCCGTGTAGTTGGTCTCTGGGGTG

Peak41742 (B9)

>C57BL/6 (chr18:37147967-37148555)

ACAGGGCAGCTGTCACTTCTCATTCCTTTTTAAGGTCGGGTATCTGTtgttattagacgcgttctcacgaccggccaggaaagacgcaacaaaccagaatcttcTGCGGCAaagctttattgcttacatcttcaggagccagagtgcaagaagcaagagagagagaaaacgaaaacccgtcccttttttaggagagttatatttcgcctaggacgtgtcactccctgattggctgcagcccatcggccgagttgacgtcacggggaaggcagagcacatggagtggagaaccaccctcggcatatgcgcgtttatttgtttaccacttagaacacagctgtcagcgccatcttgtaacggcgaatgtgggcgcggctcccaacaGTGTCCTACTCACTGGTGGAGCGGAGGGTGGGCGAGCGCTTGCTGTCGAGCTATATGTCTGTGCACGCGGAGAGCGGCAAGGTGTTCGCGCTGCAGCCTCTGGACCATGAGGAGCTGGAGCTGCTGCAGTTCCAGGTGAGCGCGCGGGATGCTGGTGTGCCTGCCCTGGGCAGCAATGTGACTCTGCAGGTGTTTGTACTCGATGAGAATGATAATGCTCCTTCATTCTTTTGGCTGGTACTGAAGGCT

>Cast/EiJ

No amplicon could be obtained from the Cast genomic DNA

**Peak93929 (B10) Some motifs disrupted**

>C57BL/6 (chr8:127306927-127307345)

ACCAGTGTTCCAGTCCCAAGCTGGGCCGGAGGGCGGAGCCTTCTCCTGATTTATCCACTGGAGTGCCCTTGTTTACTTAGTCTCAGTGCCAAGGACCTACTCCTGTCCACACACTGCCTGAAACCACAGC.TAGCATAGCCTCTACTCTCCAAATGGCGTTTTCTTGTCAGGAAACAAATCACAGAAAAGCAAACCCTGAAAGGCAGAACAGTGGGCTATGAAAAGTCCCTAAGGGATACAAGCATTTGCTCTCTTCGCCTTACTGAGACAGATA**T**CGAGAAAACAGCAGCAGTAGAAAGGAACTGATC**T**CAGCACTCGGCTCCCAC**A**GCACA**TGCGGCA**CA**TGCGGCA**CAGCAGCG**A**TAGCACGAGGCAGCAGAGACAGCGGGTCACGTGGCATCCACAGACCGGAAGCAGCTAGTCA

>Cast/EiJ

ACCAGTGTTCCAGTCCCAAGCTGGGCCGGAGGGCGGAGCCTTCTCCTGATTTATCCACTGGAGTGCCCTTGTTTACTTAGTCTCAGTGCCAAGGACCTACTCCTGTCCACACACTGCCTGAAACCACAGC**C**TAGCATAGCCTCTACTCTCCAAATGGCGTTTTCTTGTCAGGAAACAAATCACAGAAAAGCAAACCCTGAAAGGCAGAACAGTGGGCTATGAAAAGTCCCTAAGGGATACAAGCATTTGCTCTCTTCGCCTTACTGAGACAGATA**G**CGAGAAAACAGCAGCAGTAGAAAGGAACTGATC**C**CAGCACTCGGCTCCCAC**G**GCACA**TGCGGCA**CA**GCA**GGCACAGCAGCG**G**TAGCACGAGGCAGCAGAGACAGCGGGTCACGTGGCATCCACAGACCGGAAGCAGCTAGTCA

**Peak85736 (B11) false positive SNP, peak removed**

>C57BL/6 (chr7:134755064-134755738)

CTTGATGAGCGTGGAGCTGTGGCCGAAACTCTTGCCACACTCCAGGCACTCGTAGGGCTTCTCCCCGGTGTGTGTGCGCTGGTGCTGCGTCAGCTCCGAGCTCTGGATGAAACTCT**TGCCGCA**CTCGGTGCAGCGATAGGGCTTCTCGCCTGCATGGATCTTCTGGTGCTTGAGCAGGTTATGATTCTGGCCAAAGCGCT**TGCCGCA**CTCCGGGCACTTGTAGGGCTTCTCACCTGTGTGCGTGGCCTGGTGCTGGATGAGGTCCGAGCTGCGGTAGAAAGCACGTCGGCACTCGCCACACTTGTAGGGCTTCTCCCCGGTGTGGGAGCGCTGGTGCTTGATGAGGTTGGTGCTTTGTGTGAAGGCtttctcgcactccgtgcacttgtagggcttctcgcccgtgtgcgtgcgctggtgctgcactagattggagctccagctgaagcacttgccacagtcggggcacttgtaaggcttctcgcctgtgtgtgtgcgctggtgCTGCACCAGGTGCGAGCTCTGCGTGAAGCTCT**TGCCGCA**CTCTGAGCACGTGTTGGGCCGCTCCCCCGTGTGGATTCGCTGG**TGCCGCA**GCAGTTTGGACCACTGGCTAAAGCTCTTGCCACACTC**G**TTGCAGATGTATGGCTTCTCGGCCCCAGATGGGCGTCCCTGCAC

>CAST/EIJ

CTTGATGAGCGTGGAGCTGTGGCCGAAACTCTTGCCACACTCCAGGCACTCGTAGGGCTTCTCCCCGGTGTGTGTGCGCTGGTGCTGCGTCAGCTCCGAGCTCTGGATGAAACTCT**TGCCGCA**CTCGGTGCAGCGATAGGGCTTCTCGCCTGCATGGATCTTCTGGTGCTTGAGCAGGTTATGATTCTGGCCAAAGCGCT**TGCCGCA**CTCCGGGCACTTGTAGGGCTTCTCACCTGTGTGCGTGGCCTGGTGCTGGATGAGGTCCGAGCTGCGGTAGAAAGCACGTCGGCACTCGCCACACTTGTAGGGCTTCTCCCCGGTGTGGGAGCGCTGGTGCTTGATGAGGTTGGTGCTTTGTGTGAAGGCtttctcgcactccgtgcacttgtagggcttctcgcccgtgtgcgtgcgctggtgctgcactagattggagctccagctgaagcacttgccacagtcggggcacttgtaaggcttctcgcctgtgtgtgtgcgctggtgCTGCACCAGGTGCGAGCTCTGCGTGAAGCTCT**TGCCGCA**CTCTGAGCACGTGTTGGGCCGCTCCCCCGTGTGGATTCGCTGG**TGCCGCA**GCAGTTTGGACCACTGGCTAAAGCTCTTGCCACACTC**G**TTGCAGATGTATGGCTTCTCGGCCCCAGATGGGCGTCCCTGCAC

===> Predicted SNP failed to validate experimentally and was deemed a false positive, peak 85736 was moved to the NI category. We note that this SNP has now also been removed from the Sanger database.

**Peak18283 (B12) All motifs disrupted**

>C57BL/6 (chr12:13443818-13444125)

GATCAAGAAGGGGACAGCAAC**C**GTAGCAGCAAACCAGTAGTGTGTGATGGCCGGGATGCTCCTGAACCAGTCCCCGATGTCC**G**ACATGGTCGCCCATCAGGTGACCGGTT**TTG**CAG**TGCCGCG**CGTCCTC**A**TGTGCCCAC**T**C**T**GCGCTCC**GT**GGCCGGCTTGGCTAGGGAAAGTGGAGG...........TACAGGAGGCGGAGACCGA**CT**GACGT**T**GCGCCACCGAATTCGGACCGCCATGCTGGGTCCTTAA**CCTGAA**ACCATTAGGATACTATACATTAGGATACTAGAGGAAAACTGAGGATTCTAGGCAGAGGC

>Cast/EiJ

GATCAAGAAGGGGACAGCAAC**T**GTAGCAGCAAACCAGTAGTGTGTGATGGCCGGGATGCTCCTGAACCAGTCCCCGATGTCC**A**ACATGGTCGCCCAT**T**AGGTGACCGGTT**GCA**CAG**TGCCACG**CGTCCTC**C**TGTGCCCAC**C**C**C**GCGCTCC**AC**GGCCGGCTTGGCTAGGGAAAGTGGAGG**CTCAGCCGCTG**TACAGGAGGCGGAGACCGA**TG**GACGT**G**GCGCCACCGAATTCGGACCGCCATGCTGGGTCCTTAA**TGCAAG**ACCTGAAACCATTAGGATAGTAGAGGAAAACTGAGGATTCTAGGCAGAGGC

**Peak58561 (B13) All motifs intact**

>C57BL/6 (chr4:32981052-32981892)

TGAGAGCACAGCACAGTCCTGCTTCTCT**T**CACTCTGCGGGC**G**GCTGTGTGGCTGCTCGCTCTATGCCCACTCAGAATGT**C**CTATCAGCAGCTCAGGAACCACAGGGACAAGCTTTAAAGGCCCCTCACTGAGGGCTAAGGAGATGCTGGCTCAGCTGGCAAACGGCTAGGATGAAAGCACAAAGACCTGAGTTTGAGCCTCGGCACCCAC**A**TGAAAACACA**T**GGCACGGACCGGGG**.**GATGATGG**C**CTCAGCA**TT**TAACGGCAAT**TGCCGCG**AAG**C**CCGAGTTCAAACCCAGGAAGCCACGTGATGGAAAGAATAAACCAATTCCCGAAAGTTGTCCT**T**CCCCC**T**C**AC**AC**...**ACGCACACCCCACGCATACTG**C**GGTTTGAACGAGAACGGTCCCCATTGG**C**TCATATACTTGAATGCTTAGTCACCAGGGAG**T**AGAACCATTTGAGAAGAGTTAGAA**G**GATTAGGAGGTGTGGCCTTGTTGAAGGGAGCGTGTCAC**T**GGGGCTGGGCTCTAGTTTTAAAAGCCTAAGTCAGGCCCAGGGTTTCTCTCTGCTTGCTGCCTGAAGATCAGGGTGTAAGGCTCTCAGCTCCTCCTCAGGCACCATGCCTGTCTGATTCCCACC**A**TTGACAATCATGGGTGAACCTTCTAAAACTGTAAGCAAGGGCTGGAGAGATGGCTCAGCAGTTAAGAGCACTGGGTGCTATTCTAGAGGTCTTGAGCTCAATTCCCAGCAACCACGTGGTGGCTCACAACCACCTGTAATGGGATCTGATGGCCTCTCCTGATGTGCCTGAAGACTGTCGTGTATACGTATGAATGTGTGTGTGTGTGTAGTGAGCAAGCCC

>Cast/EiJ

TGAGAGCACAGCACAGTCCTGCTTCTCT**C**CACTNTGCGGGC**A**GCTGTGTGGCTGCTCGCTCTATGCCCACTCAGAATGT**T**CTATCAGCAGCTCAGGAACCACAGGGACAAGCTTTAAAGGCCCCTCACTGAGGGCTAAGGAGATGCTGGCTCAGCTGGCAAACGGCTAGGATGAAAGCACAAAGACCTGAGTTTGAGCCTCGGCACCCAC**G**TGAAAACACA**C**GGCACGGACCGGGG**A**GATGATGG**G**CTCAGCA**GG**TAACGGCAAT**TGCCGCG**AAG**T**CCGAGTTCAAACCCAGGAAGCCACGTGATGGAAAGAATAAACCAATTCCCGAAAGTTGTCCT**C**CCCCC**C**C**CA**AC**CCC**ACGCACACCCCACGCATACTG**A**GGTTTGAACGAGAACGGTCCCCATTGG**T**TCATATACTTGAATGCTTAGTCACCAGGGAG**A**AGAACCATTTGAGAAGAGTTAGAA**A**GATTAGGAGGTGTGGCCTTGTTGAAGGGAGCGTGTCAC**C**GGGGCTGGGCTCTAGTTTTAAAAGCCTAAGTCAGGCCCAGGGTTTCTCTCTGCTTGCTGCCTGAAGATCAGGGTGTAAGGCTCTCAGCTCCTCCTCAGGCACCATGCCTGTCTGATTCCCACC**G**TTGACAATCATGGGTGAACCTTCTAAAACTGTTAGCAAGGGCTGGAGAGATGGCTCAGCAGTTAAGAGCACTGGCTGCTATTCTAGAGGTCCTGAGCTCAATTCCCAGCAACCACGTGGTGGCTCACAACCACCTGTAATGGGATCTGATGGCCTCTCCTGATGTGCCTGAAGACTGTCGTGTATACGTATGAATGTGTGTGTGTGTGTGTGTGTGTGTGTGTGTGTGTGTGTGTGTGTAGTGAGCAAGCCC

**Peak64075 (B14) All motifs intact**

>C57BL/6 (chr4:140379533-140379955)

AGGGTTGTACCACCATGCTCCACACCCACGTGCCTGGCACGTGACCTTAATGTGAATGAACAAACTGACCACTGACAATT**TGCCGCA**CACCGTGGTGACTGCGGCTGAGCAGCACAGCTCACATTTGTGGGCATCGGACTCCAGAGACTCACAGCTCCCTCCCTGAAGCCCTCCAGGCCTGAAGCCCAGGCTGTGGTAGGA**C**CAGC**C**CCTGTA**C**GCTATCTGAGATGGGTGTCCGGCAGGGTCACCCCCAGC**T**ACACTCA**A**CCCAGTACAGCAGGC**CGCGGCA**CCCAGCTGTGTGCCTCCCTTGGTACCAGCAACCTCTCCCTTGT.ATG**A**TTTCACAGC**C**TTGGGCTTTCCACCTGAG**G**CTATGTGACCTT**T**C**T**ACTGTCAGAGGCCAGCTGGGCCTCAGAGGATGAGTTAGGGCACACCCAGA

>Cast/EiJ

AGGGTTGTACCACCATGCTCCACACCCACGTGCCTGGCACGTGACCTTAATGTGAATGAACAAACTGACCACTGACAATT**TGCCGCA**CACCGTGGTGACTGCGGCTGAGCAGCACAGCTCACATTTGTGGGCATCGGACTCCAGAGACTCACAGCTCCCTCCCTGAAGCCC**................**AGGCTGTGGTAGGA**T**CAGC**T**CCTGTA**T**GCTATCTGAGATGGGTGTCCGGCAGGGTCACCCCCAGC**C**ACACTCA**G**CCCAGTACAGCAGGC**CGCGGCA**CCCAGCTGTGTGCCTCCCTTGGTACCAGCAACCTCTCCCTTGTA**A**TG**T**TTTCACAGC**T**TTGGGCTTTCCACCTGAG**T**CTATGTGACCTT**C**C**C**ACTGTCAGAGGCCAGCTGGGCCTCAGAGGATGAGTTAGGGCACACCCAGA

**Peak8617 (B15) All motifs intact**

>C57BL/6 (chr10:84966229-84966624)

GGGACAAGGGTCTGTGAGAAGCAAGACACAAACACCACACACACACACACACACACACACAC**G**CACAC**G**CGCATGAACCCTTGCAGGGCTGGAGGACCTGGTTCCAGAGCCAGCCTGCCTCATGTGTGAAAACTGACCCTGCTGCCAACCCACATTCTGTGGCCACATCC**C**GCTCTGCTCTGTGCGG**CGCGGCA**AGTCTTCTTGTTCTTTTGTGTGCACATGTAAACCCCAGAAAACTCTGGAAACCACAGGATTATTTTTCCCACATAGTCAGCACAAATTCACTCAACAGCTTATCCTAATCAAAACACAAGCTCCTCTCTCTCTCTCTCTCT**T**TCTCTCTCTCTCTCTCTCTCTCTCTCT......GAACAACACCGCATCCTGCTAAGGCTGTTGCTT

>Cast/EiJ

GGGACAAGGGTCTGTGAGAAGCAAGACACAAACACCACACACACACACACACACACACACAC**A**CACAC**A**CGCATGAACCCTTGCAGGGCTGGAGGACCTGGTTCCAGAGCCAGCCTGCCTCATGTGTGAAAACTGACCCTGCTGCCAACCCACATTCTGTGGCCACATCC**A**GCTCTGCTCTGTGCGG**CGCGGCA**AGTCTTCTTGTTCTTTTGTGTGCACATGTAAACCCCAGAAAACTCTGGAAACCACAGGATTATTTTTCCCACATAGTCAGCACAAATTCACTCAACAGCTTATCCTAATCAAAACACAAGCTCCTCTCTCTCTCTCTCTCT**C**TCTCTCTCTCTCTCTCTCTCTCTCTCTCTCTCTGAACAACACCGCATCCTGCTAAGGCTGTTGCTT

**Peak42700 (B16) Some motifs disrupted**

>C57BL/6 (chr18:74520155-74520615)

TTTGCCCTATTCCGAGTTTGGAACATCCCAGGGCAATTTAAACTCACCCCTTATGAGATCCTGTGTAGGGGACCACTGTGGAGAGCCG**TGCCGCG**AGCAATCG**T**GTGCG**TGCCGCA**AGGAATTGCTGAGGAGAGCCGTGTGTGCCATGAGCAATCGCCATTATAAGATGGCGCTGGCCTCCGCTGTGCCTAACTAGTAAACAAGCCTTGTACGCAGGT**G**CGAGAGTGAACTCACTCCTAGTCACTGCCCATTCTCGGGGCGTAATAGTGGGGTGATGGGCAAGCAACAAATCAGGAGCTGTCACGCCACATCAGGTGCTGAAACGTCACGCTGCGGGCTATATAAGCAGCGCCATTTTCCCGGTTCGGGGTCTTCCTGAAGAAGTAAGCAATAAAGCTTT**TGCCGCA**GAAGATTCCGGTGGTTGCGTCTTTCTTGCCGGT**C**GAGTGGGACGCAATAAGTGG

>CAST/EiJ

TTTGCCCTATTCCGAGTTTGGAACATCCCAGGGCAATTTAAACTCACCCCTTATGAGATCCTGTGTAGGGGACCACTGTGGAGAGCCG**TGCCGCG**AGCAATCG**C**GTGCG**TCCTGCA**AGGAATTGCTGAGGAGAGCCGTGTGTGCCATGAGCAATCGCCATTATAAGATGGCGCTGGCCTCCGCTGTGCCTAACTAGTAAACAAGCCTTGTACGCAGGT**A**CGAGAGTGAACTCACTCCTAGTCACTGCCCATTCTCGGGGCGTAATAGTGGGGTGATGGGCAAGCAACAAATCAGGAGCTGTCACGCCACATCAGGTGCTGAAACGTCACGCTGCGGGCTATATAAGCAGCGCCATTTTCCCGGTTCGGGGTCTTCCTGAAGAAGTAAGCAATAAAGCTTT**TGCCGCA**GAAGATTCCGGTGGTTGCGTCTTTCTTGCCGGT**T**GAGTGGGACGCAATAAGTGG

Peak233 (B17)

>C57BL/6 (chr1:24602139-24602558) Cast sequence absent

GGTCGCTATCTCCGTGGTAAGTGCCAGTGGGATCAATGCCATGCTTGTCGCTGATTACCTCCCAGAACTTGGCGCCAATCTGGTTGCCGCAGTGCCCAGCCTGCAGGTGCATGATCTCCCTCATGGCGGCAGCCGCCGCGGAACAGTTGTAGAAGAGGAGCACTTCGGGAGGAGAAGGCGcacagagtccccatgcctcttcttacccctcccatttgtctctataagggtgctcctctacccactcatccactccagcctcacctctctagcatctccctccctatgatgcggcatcaaacctccacaggatcaagggtatcccctcccactgatgtcagacaggtccatcctctgctacatatgtgtctggatccctggatccctccatatgtactcattggttggcagtttaatccc

>Cast/EiJ

No amplicon could be obtained from the Cast genomic DNA

Peak89880 (B18) Failed to amplify Cast genome-specific sequence

>C57BL/6 (8:79972368-79973470)

ACCTGCTGCAGACACACAGAGAtgtgtagaaaggccaaaggttaacaagcagctgtcctcctcagcagctgtcttcctctgttccctttctccaccttcatttctcctcagacagagcctcactgaacctggagctcggctcctcagtcagactctctagctggaagccccaggatgtccctggcttccctctctggtgcaaggctacctgtgcacacatccctagcCCTGTGCACACTGCCGCATCCCTGGCCCTGTGCACACTGCCGCATCCCTGGCCCTGTGCACACTGCCGCATCCCTGGCCCTGTGCACACTGCCACATCCCTAGCCCTGTGCACACTGCCGCATCCCTGGCCCTGTGCACACTGCCACATCCCTGGCCCTGTGCACACTGCCACATCCCTGGCCCTGTGCACACTGCCACATCCCTGGCCCTGTGCACACTGCCGCATCCCTGGCCCTGTGCACACTGCCGCATCCCTGGCCCTGTGCACACTGCCGCATCCCTGGCCCTGTGCACACTGCCGCATCCCTGGCCCTGTGCACACTGCCGCATCCCTGGCCCTGTGCACACTGCCGCATCCCTGGCCCTGTGCACACTGCCACATCCCTGGCCCTGTGCACTCTGCCGCATCCCTAGCCCTGTGCACACTGCCACATCCCTGGCCCTGTGCACACTGCCGCATCCCTGGCCCTGTGCACACTGCCACATCCCTGGCCCTGTGCACACTGCCACATCCCTGGCCCTGTGCACACTGCCACATCCCTGGCCCTGTGCACACTGCCACATCCCTGGCCCTGTGCACACTGCCGCATCCCTGGCCCTGTGCACACTGCCGCATCCCTGGCCCTGTGCACACTGCCGCATCCCTGGCCCTGTGCACACATCCCTGGCcctgtgcacactgccgcatccctggccctgtgcacactgccgcatccctagccttcttacatgggttgggggaagccaaacttgaatcattatgcttgtatggcaggcacattaccaaatgagacatcttctcagctccC]TCCATGCAATTCTTAACATCTAGAAAATATCTCAGAATGTAGGGCACAGTGCAA

>Cast/EiJ

No amplicon could be obtained from the Cast genomic DNA

**Peak85736 (C1) All motifs intact**

>C57BL/6 (chr7:134379391-134379818)

GGACAGTTCAGCCCGAATAAAGCACCAGCGGACACACAGAGGGGATCAGCTGCCCCGGCCAGTGGTTCCCCGACGGCAGCCATCCCCGGCAGCTCCGGCGGCTCCACACAGACCCAAGGCCCAGGACAAGCCGTATATCTGCACCGAC**TGCGGCA**AAAGGTTTGTGCTCAGCTGCAGCCTGCTGAGCCACCAACGGAGTCACTTAGGCCCCAAACCCTTCGGTTGTGATGTGTGTGGAAAGGAGTTTGCGCGGGGCTCTGACCTGGTGAA**A**CATCTGCGGGTACACACAGGTGAGAAGCCCTACCTGTGCCCTGAGTGTGGCAAAGGCTTTGCTGATAGCTCCGCCCG**G**GTGAAGCA**T**CTCCGCACCCACAGCGGCCAGAGGCCTCACGCCTGCCCAGAGTGTAACCGTAGCTTTAGCCTCAGCTCCA

>Cast/EiJ

GGACAGTTCAGCCCGAATAAAGCACCAGCGGACACACAGAGGGGATCAGCTGCCCCGGCCAGTGGTTCCCCGACGGCAGCCATCCCCGGCAGCTCCGGCGGCTCCACACAGACCCAAGGCCCAGGACAAGCCGTATATCTGCACCGAC**TGCGGCA**AAAGGTTTGTGCTCAGCTGCAGCCTGCTGAGCCACCAACGGAGTCACTTAGGCCCCAAACCCTTCGGTTGTGATGTGTGTGGAAAGGAGTTTGCGCGGGGCTCTGACCTGGTGAA**G**CATCTGCGGGTACACACAGGTGAGAAGCCCTACCTGTGCCCTGAGTGTGGCAAAGGCTTTGCTGATAGCTCCGCCCG**C**GTGAAGCA**C**CTCCGCACCCACAGCGGCCAGAGGCCTCACGCCTGCCCAGAGTGTAACCGTAGCTTTAGCCTCAGCTCCA

**Peak15960 (C2) Some motifs disrupted**

>C57BL/6 (chr11:113715206-113715579)

TAGACACGTCAGGCTTGCAGCAGGACATTCCGGGGTTGTG**A**GGGAGGTGGGCTGTTG**CA**GTCTAGTCACCTCTTGCTTCCTG**C**CTGCTCCCACAGCAGC**T**CT....**.......**.....GGCC**TGCGGCA**GCCCTGGCC**TGCAGCA**GCCCTGGCTTCCATGCAGCCTGC**G**GGGATTAAACTGTGCCCTTGCT**G**GGCTGGTCCT**A**CT**CA**TTTTCAAGGCTATTGGTGTTGTCCGTTCTTCTTTGGCCATTTTACCGTAGCCCTTCTTGTCGCAGTGTGGGCATTTCTCATCACCTGGTCTTTATGTCGGCAAGGATGGACCTTGAGTCTGTACTGTTCTCTAAGCCCCTCCCCCC**.**TCAGGGTAGGAAGCCCTGATGGTCCTAGAATGGCCCT

>Cast/EiJ

TAGACACGTCAGGCTTGCAGCAGGACATTCCGGGGTTGTG**G**GGGAGGTGGGCTGTTG**AG**GTCTAGTCACCTCTTGCTTCCTG**T**CTGCTCCCACAGCAGC**C**CT**AGCCTGCGGCAGCCCT**GGCC**TGCGGCA**GCCCTGGCC**TGCGGCA**GCCCTGGCTTCCATGCAGCCTGC**A**GGGATTAAACTGTGCCCTTGCT**A**GGCTGGTCCT**T**CT**AG**TTTTCAAGGCTATTGGTGTTGTCCGTTCTTCTTTGGCCATTTTACCGTAGCCCTTCTTGTCGCAGTGTGGGCATTTCTCATCACCTGGTCTTTATGTCGGCAAGGATGGACCTTGAGTCTGTACTGTTCTCTAAGCCCCTCCCCCC**C**TCAGGGTAGGAAGCCCTGATGGTCCTAGAATGGCCCT

**Peak5656 (C3) Some motifs disrupted**

>C57BL/6 (chr10:43608385-43608997)

CCATGATTTCAGCTCAGCAACAATGAACTAAAGCAGGCAGCATTCACATAGCGAATGAATGCCAGAATTAGTAAAAAAATAAGATTCATGAAGCCTGCTCATGCTCAATGTTTTATTTGGTAAGATCCATAAAGCCATGCTTTAAAATAACCAGAGATTACAGGAGAA**A**ATTTTAAATGTCATTTTAAGTTAAGTTTTTAATGTACAAAGTTCACATCGTGGGCTAAAGTTGAGTACCCTTTGAGGTCAATTCCATATATTTTTGAACACAAACAGCTCACCAAAAAGAAATACAGAGACCAGAGTCTGAGTTCCTGTGCCACTGGGCTTTGCATGTATTTAGACCATGTGTATCTGGGCAGGGCTTAAAGCAGACAGAAAGCATCTTACGCAAAGCAGGTGAACGCGGCCACGGC**TGCTGCG**C**TGCCGCC**CGAGC**TTCCGCC**AGTGATCAGCCAGTGGGAATCATCACCTGCATCCTGC**C**GGCTTCTCTCTCTGTACTGCTTCGAGTAAGTCCAGGGGTTTCTAACTGGCCCAAACACACCATCTGTGCTTCCAGACCTGCGACGGAGAGACAAAGCAGTGCCTCAGAAAGACTTCTTATGTCCCAGCGAGC

>Cast_C1

CCATGATTTCAGCTCAGCAACAATGAACTGAAGCAGGCAGCATTCACATAGCGAATGAATGCCAGAATTAGTAAAAAAATAAGATTCATGAAGCCTGCTCATGCTCAATGTTTTATTTGGTAAGATCCATAAAGCCATGCTTTAAAATAACCAGAGATTACAGGAGAA**C**ATTTTAAATGTCATTTTAAGTTAAGTTTTTAATGTACAAAGTTCACATCGTGGGCTAAAGTTGAGTACCCTTTGAGGTCAATTCCATATATTTTTGAACACAAACAGCTCACCAAAAAGAAATACAGAGACCAGAGTCTGAGTTCCTGTGCCACTGGGCTTTGCATGTATTTAGACCATGTGTATCTGGGCAGGGCTTAAAGCAGACAGAAAGCATCTTACGCAAAGCAGGTGAACGCGGCCACGGC**TGCCGCG**C**TGCCGCC**CGAGC**TGCCGCC**AGTGATCAGCCAGTGGGAATCATCACCTGCATCCTGC**T**GGCTTCTCTCTCTGTACTGCTTCGAGTAAGTCCAGGGGTTTCTAACTGGCCCAAACACACCATCTGTGCTTCCAGACCTGCGACGGAGAGACAAAGCAGTGCCTCAGAAAGACTTCTTATGTCCCAGCGAGC

**Peak155 (C4) Some motifs disrupted**

>C57BL/6 (chr1:16590898-16591324)

AGTGTGCATGTGGACCATGTGTGAGCAGAGGT**A**TGCATGCCAAGGTGTGTAGCCATGGTCAGGGGACAGCTTTTTGAAGCTGTTTCTCACCTTCTACCTTTACATAGGTTCTGAAGATCAAACTCACATCACCAGGCCTG**TGCGGAA**AACACCCTT**AGCGGCA**CAGTTTCTCAGCAGTCTCAGTCCCACAGCCTGTGCCATGACTGCAATCGTGGAGTGTGCGCCTTGGAGTAAGGATGTAATGGGAAGTCAGCTTATCT**T**TCTCCAAAGAGTCTCAACTTTTGAACTCCTATAACTACTACTTTTT**T**AAAAAAGGTAATTTGGTTTTTTTCACACATCTTCACTTCTAATAAAGAAATAGCTCCGTACTCTACTCCAGAGAAGAGCTAGGTTTATGGACTAAGACAAGCAGCATGCAAACACTGTC

>Cast/EiJ

AGTGTGCATGTGGACCATGTGTGAGCAGAGGT**G**TGCATGCCAAGGTGTGTAGCCATGGTCAGGGGACAGCTTTTTGAAGCTGTTTCTCACCTTCTACCTTTACATAGGTTCTGAAGATCAAACTCACATCACCAGGCCTG**TGCGGCA**AACACCCTT**AGCGGCA**CAGTTTCTCAGCAGTCTCAGTCCCACAGCCTGTGCCATGACTGCAATCGTGGAGTGTGCGCCTTGGAGTAAGGATGTAATGGGAAGTCAGCTTATCT**C**TCTCCAAAGAGTCTCAACTTTTGAACTCCTATAACTACTACTTTTT**A**AAAAAAGGTAATTTGGTTTTTTTCACACATCTTCACTTCTAATAAAGAAATAGCTCCGTACTCTACTCCAGAGAAGAGCTAGGTTTATGGACTAAGACAAGCAGCATGCAAACACTGTC

**Peak41084 (C5)**

>C57BL/6 (chr18:10044188-10044938)

ggaagagaaggcagctctcacagctggctgcactgggccaacttggggaagggggcggaggggaatgtggtggaggcagcaactctagtcaaccctagaaagccaggaggggccagcgtgggccctgtgggcctcacccctctttcttttcttgcccgttccgcaggtggtgaaatgtatggcggtggtggagcaggcatcccggggaaacctccacatcagtgttccccccaggccacagtttctcacgtgacacctcgccttggcaagtgcacgctgctgctgccgccgaagccggaagttctcagtccggtcctgcggctgtgttcagccagggatgtgcagagttgtttttcttaactatcactgacaaattctgtcctaatgtaagtccaagtccacagtcggcaccacacacacagtctgtcccatgacaaaaaccacaaacaaaaacaagacagcaacacaaaaacaagacagcaacagagaataaaagacaccagtatctaagcactcattcctcactctgccagatgtaaaaccaaggcacgaggtgggactcgaacccacgatctcgtaaccctaaaaccaagacacaaattcacaaaccaagacacaaatttacaaactaagacacaaattcataaaccaagacacaaatttacaaaccaagacacaaatttagtggtgccacacttagacaacggacaacatgtaagacgcacacaaacaaacgtacctccaaggggtc

>Cast/EiJ

No amplicon could be obtained from the Cast genomic DNA

**Peak16240 (C6) no motifs found**

>C57BL/6 (chr11:115302232-115302795)

TCCTGGTTCCCTATGGTCAGACATCTGCTTCCAAAAGAAAGATGTAAAAAGGAGTATGCCTATGACATAAAGAACATGCCCCTGATGTGCAAGACCATGTCCCTGGGGTAAAGGGCCATGCCCTGGCCCCCTGCCATGCCCGGATTTTTCTCTGTCTTCTCAGGATCAGCCCAGGACAAACCCCCCTCACAGCTCTCTTCCCAGCCCTCCAGAACCCCGCTCCATCTCCTGATGGGCAGCAACAGCAGTCAGCACATGTTGGAAGACCAAGTCCTCTGTCCCATTTGCCTGGAGGTGTTC**T**GCAACCCAGTCACTACCGCCTGCGGCCATAACTTCTG**T**ATGACTTGCCTTCAGAATTTCTGGGACCACCAGGCTGCCATTGGAGAGACTTACTATTGCCCCCAGTGCAGAGAGGCCTTCTCCAGCAGGCCCCGCCTCTGCAAGAACGTCATCCTCGGGGAGATGGTGGCTTGCTTCACCCAGGCCAAGAGCCAGACCTCAGGGTCCTTGTGGGGCCTGGCTGGCCCCACAGATGTGCCCTGTGACTTTTGCTCTCCGCAGAAG

>Cast/EiJ

TCCTGGTTCCCTATGGTCAGACATCTGCTTCCAAAAGAAAGATGTAAAAAGGAGTATGCCTATGACATAAAGAACATGCCCCTGATGTGCAAGACCATGTCCCTGGGGTAAAGGGCCATGCCCTGGCCCCCTGCCATGCCCGGATTTTTCTCTGTCTTCTCAGGATCAGCCCAGGACAAACCCCCCTCACAGCTCTCTTCCCAGCCCTCCAGAACCCCGCTCCATCTCCTGATGGGCAGCAACAGCAGTCAGCACATGTTGGAAGACCAAGTCCTCTGTCCCATTTGCCTGGAGGTGTTC**C**GCAACCCAGTCACTACCGCCTGCGGCCATAACTTCTG**C**ATGACTTGCCTTCAGAATTTCTGGGACCACCAGGCTGCCATTGGAGAGACTTACTATTGCCCCCAGTGCAGAGAGGCCTTCTCCAGCAGGCCCCGCCTCTGCAAGAACGTCATCCTCGGGGAGATGGTGGCTTGCTTCACCCAGGCCAAGAGCCAGACCTCAGGGTCCTTGTGGGGCCTGGCTGGCCCCACAGATGTGCCCTGTGACTTTTGCTCTCCGCAGAAG

**Peak83055 (C7) All motifs disrupted**

>C57BL/6 (chr7:54308599-54308981)

GCTGGGGACCAGTTCTGTAA**A**GGAGAGTGTGAGAGTGTGAGTTTAAAACTATGGCTGTTCTTACCCCCTCTTAGGCGCCCGGGGGAAAGCTGCTGGGTTATCCCCAGTCCCCTTCTCTCAAGCTCTCAAGTACTCTTTAGCCACAAACCTTCCTTTAGCAGTCTTTATCCTCTCGCCCTTTCAGGTCTCCTGATGTTTGCCGTGGCACACATCCTCTA**CGCCGCG**GCCTTTGGCATGCGGCCACTGGCTCTGCGGACAGGCCTGGTGATCGGAGTGCTGTCAGGCCTGTGCTATGCCCTGCTCTACCCTGGCCTGTCAGGTGCTTTCACCTACCTGGTGGGGGTCTATGTGGCCCTCATCAGCTTCATGGGCTGGAGAGCTAT

>Cast/EiJ

GCTGGGGACCAGTTCTGTAA**G**GGAGAGTGTGAGAGTGTGAGTTTAAAACTATGGCTGTTCTTACCCCCTCTTAGGCGCCCGGGGGAAAGCTGCTGGGTTATCCCCAGTCCCCTTCTCTCAAGCTCTCAAGTACTCTTTAGCCACAAACCTTCCTTTAGCAGTCTTTATCCTCTCGCCCTTTCAGGTCTCCTGATGTTTGCCGTGGCACACATCCTCTA**TGCCGCG**GCCTTTGGCATGCGGCCACTGGCTCTGCGGACAGGCCTGGTGATCGGAGTGCTGTCAGGCCTGTGCTATGCCCTGCTCTACCCTGGGCTGTCAGGTGCTTTCACCTACCTGGTGGGGGTCTATGTGGCCCTCATCAGCTTCATGGGCTGGAGAGCTAT

**Peak51244 (C8) no motifs found**

>C57BL/6 (chr2:130830541-130830938)

AAGCCTGAGGACATGCAGTTCATCTGCAGCCTCTCATACAAAGCGGGACACGCAGGGTGTGCCTGTCACC**T**C**A**GCACTGGACA**T**GCAGTGTGTGCCTGTCACCCCAGCACAGGACACGCAG**G**GTGTGCCTGTCACC**T**CAGCACTGGACA**T**GCAGTGTGTGCCTGTCACCCCAGCAC.......................................................................................................................................................................................................................................AGGACACGCAGTGTGTGCCTGTCACCCCAGCACTGGACACGCAGTGTGTGCCTGTCACCCCAGCACTGGGAAGCAGGGGACAGAAAGATCTTGCTTGCTGGCCAGCCACTCAAAGCTGGATCTGTGAGTTCTAGATTCAGTTAGAGACCCTGTCTCAAGTAAAATAAGGTAGAGAGGAATTGAGGAAGACACCTGATTACCTCTGGCTTCTGTATGCATGTG

>Cast/EiJ

AAGCCTGAGGACATGCAGTTCATCTGCAGCCTCTCATACAAAGCGGGACACGCAGGGTGTGCCTGTCACC**G**C**A**GCACTGGACA**C**GCGGTGTGTGCCTGTCACCCCAGCACAGGACACGCAG**T**GTGTGCCTGTCACC**C**CAGCACTGGACA**C**GCAGTGTGTGCCTGTCACCCCAGCAC**TGGACACGCAGTGTGTGCCTGTCACCCCAGCACTGGACACGCAGTGTGTGCCTGTCACCCCAGCACTGGACACGCAGTGTGTGCCTGTCACCCCAGCACTGGACACGCAGTGTGTGCCTGTCACCCCAGCACTGGACACGCAGTGTGTGCCTGTCACCTCAGCACTGGACACGCAGTGTGTGCCTGTCACCCCAGCACTGGACACGCAGTGTGTGCCTGTCACCCCAGCAC**AGGACACGCAGTGTGTGCCTGTCACCCCAGCACTGGACACGCAGTGTGTGCCTGTCACCCCAGCACTGGGAAGCAGGGGACAGAAAGATCTTGCTTGCTGGCCAGCCACTCAAAGCTGGATCTGTGAGTTCTAGATTCAGTTAGAGACCCTGTCTCAAGTAAAATAAGGTAGAGAGGAATTGAGGAAGACACCTGATTACCTCTGGCTTCTGTATGCATGTG

**Peak85934 (C9) All motifs intact**

>C57BL/6 (chr7:136141411-136141785)

TCAAACACCATGGGGACATAACTCCCAGGGTAGGC**A**TCCTTGGCGAACACATGCATCAGC**A**CTGTCT**TGCCGCA**CTCCGTGTCCCCCACCACT**G**TGATCTTGCAGC**A**GCCACTCTGCCC**T**TCCATGGTCCCGGCT**T**CGCGCCGGCCCCCCACGC**T**GCCCCC.TGCC**A**GGCACA**GGCGGCA**CCTCTGCCACCTCCTCCCCCACCGCTGCAGCTGCAGTGATGGCCCAGCTGATAAAAACACGTGCTGTTCTTCCAGAAGACCAAGCCTTGATTTTCTGCA**T**CCATATTGAACAGCTCACAACCTCTTATAACTTCAGCTCTGGGGATCGAACACCGTTTCTTCTGGCTTCTGCAGGTACCTGCACAAATGTGGCATA

>Cast/EiJ

TCAAACACCATGGGGACATAACTCCCAGGGTAGGC**G**TCCTTGGCGAACACATGCATCAGC**G**CTGTCT**TGCCGCA**CTCCGTGTCCCCCACCACT**A**TGATCTTGCAGC**G**GCCACTCTGCCC**C**TCCATGGTCCCGGCT**A**CGCGCC**A**GCCCCCCACGC**C**GCCCCC**C**TGCC**G**GGCACA**GGCGGCA**CCTCTGCCACCTCCTCCCCCACCGCTGCAGCTGCAGTGATGGCCCAGCTGATAAAAACACGTGCTGTTCTTCCAGAAGACCAAGCCTTGATTTTCTGCA**C**CCATATTGAACAGCTCACAACCTCTTATAACTTCAGCTCTGGGGATCGAACACCGTTTCTTCTGGCTTCTGCAGGTACCTGCACAAATGTGGCATA

Summary (Relates to Figure 4B):

ALL motifs disrupted 5

SOME motifs disrupted 8

ALL motifs intact 7

No motifs 2

No Cast amplicon 4

TOTAL 26
