# Supplementary material for: Allele-specific binding of ZFP57 in the epigenetic regulation of imprinted and non-imprinted monoallelic expression
Source: Genome Biol. 2015 May 30;16(1):112. doi: 10.1186/s13059-015-0672-7 (PMC4491874; doi:10.1186/s13059-015-0672-7)
Supplement: Additional file 4: — Supplementary methods. [file 13059_2015_672_MOESM4_ESM.docx]

**Supplementary Methods**

*Experimental identification of SNP and indel regions*

In order to experimentally validate genetic sequence variants between the two mouse strains we designed PCR primers spanning selected allele-specific ZFP57 binding sites. We then used these to amplify the corresponding regions from genomic DNA isolated from pure C57BL/6J and Cast/EiJ mice. The PCR products were gel-purified, A-tailed and cloned into a pGEM-t easy vector (Promega). At least one BL6 and two Cast colonies were picked from each region and subjected to Sanger dideoxy sequencing (Source Bioscience). The sequences were aligned to the reference genome and SNP/indel positions were inferred with respect to the ZFP57 binding motifs (Additional file 3). For four regions associated with non-imprinted monoallelic ZFP57 binding we could not establish an SNP list due to an inability to obtain a Cast-specific PCR amplicon. Primer sequences used to amplify genomic DNA are listed in Additional file 5: Table S2.

*Allele – specific ChIP-seq read alignments and SNP filtering*

The reads were aligned to the mm9 assembly of the mouse genome using an allele-specific alignment pipeline (ASAP; http://www.bioinformatics.babraham.ac.uk/projects/ASAP/) utilising Bowtie short read aligner software [1]. Briefly, each read was simultaneously aligned to BL6 and Cast genome sequences and best alignments for each genome were reported. Sequences that aligned equally well to both genomes were put into common alignments, others were partitioned into either BL6 or Cast genome specific alignments depending on the number of mismatches to the two genome sequences. Sequences having more than one chromosomal alignment (non-unique) were discarded and up to a maximum of one mismatch (i.e. sequencing/base calling error) was tolerated. The *Mus musculus cast* genome was constructed by injecting 20,443,889 SNPs into the BL6 reference genome. SNPs were sourced from the Biomart *Mus musculus* variation database (dbSNP128, Ensembl) (6,270,609) and further complemented by 17,259,893 high-confidence flagged SNPs in the Sanger mouse strain database [2] (data was downloaded from the Mouse Genomes Project page (http://www.sanger.ac.uk/resources/mouse/genomes/). We excluded SNPs close to indels, repeat element or those having two possible base calls for each genome. In order to reduce potential biases towards the reference BL6 genome assembly arising from falsely annotated SNPs, a condition was applied where each SNP had to have at least one BL6- and one Cast- specific read being aligned to it from across all four sequenced libraries (2 ChIPs & 2 inputs). As a result we obtained a close to 1:1 ratio of total BL6 and Cast reads in each sample (Table 1).

*Definition of the overall list of ZFP57 binding sites*

For identification of a total number of ZFP57 sites in the genome, we performed model based analysis of ChIP-seq (MACS) [3] on BC and CB ChIP data using their corresponding inputs as a controls. We used a combined set of BL6, Cast and common reads for each sample as at this stage we were not interested in the allele specificity of each peak. Closely spaced peaks were separated using a *post hoc* peak splitter tool (http://www.ebi.ac.uk/bertone/software.html). The common list of peaks from BC and CB ChIP-seq experiments were then overlapped using BEDTools [4] as shown in Additional file 1: Figure S3A. This list was next analysed using SeqMonk software (http://www.bioinformatics.babraham.ac.uk/projects/seqmonk/). Read count densities were then calculated for each peak normalising for peak length and a total number of aligned reads in each dataset (Additional file 2: Table S1, columns E and G). A total of 55,273 candidate peaks were initially selected having a mean read density equal to or greater than five. In order to make valid comparisons between BC and CB ChIP-seq experiments, we calculated percentile ranks for each peak (Additional file 2: Table S1, columns F and H) and took the average of the two (Additional file 2: Table S1, column I). The peaks were sorted according to their average percentile rank across the two experiments and subsets of peaks from different ranking values were selected for independent ChIP-qPCR validation. An experimentally derived threshold for the mean percentile rank at top1% levels was selected based on the lowest false positive rate in this category (Additional file 3, part B). Peaks over simple DNA repeats and those lacking binding motifs were not considered due to an inability to experimentally validate binding. The final list of 158 sites includes peaks in the top 1% category plus all sites experimentally validated outside this threshold.

*Definition of allele - specific peaks*

Allele – specific assignments to each of the above defined 158 peaks were made by applying a two tailed Fishers’s exact test to genome- specific reads underneath each peak. Briefly, for each peak a 2 by 2 contingency table was constructed testing separately for significant read skews towards either parental or genetic origin of the allele. For example:

Test for parental origin effects, peak 83234 (Snrpn ICR):

|  | BxC ChIP | CxB ChIP |
| --- | --- | --- |
| BL6 reads | 95 | 1 |
| Cast reads | 6 | 78 |

p= 1.64E-42

|  | BxC ChIP | CxB ChIP |
| --- | --- | --- |
| Mat. reads | 5 | 34 |
| Pat. reads | 91 | 2 |

Test for genetic origin effects, peak 85736 (Cast- specific):

p= 8.15E-24

P-value for parental and genetics effects were calculated for each peak and are listed in Additional file 2: Table S1, columns R and S respectively.

*Feature overlap analysis*

Overlap analysis of Zfp57 peaks with various genomic features was performed using BEDTools (intersectBED function) suite [4]. Mouse Ensembl (mm9) gene annotation was downloaded from Biomart central server, promoter regions were taken as -1.5 to +0.5 kb regions from the transcription start site (TSS). Repeat element coordinates were downloaded from the UCSC repeat masker via Galaxy interface (<https://main.g2.bx.psu.edu/>). In order to generate a control boot-strap set, Zfp57 peak coordinates were randomly shuffled across the genome using BEDTools (shufflebed function) and overlapped with each of the genomic features. The process was repeated 9 more times and an average number of overlaps with each type of feature was taken and shown in Figure 2A top graphs. These represent the genome average occurrence of these features if Zfp57 binding was random.

*Histone modification analysis*

Genomic coordinates for enrichment of H3K4me3, H3K27me3, H3K9me3 and H4K20me3 were taken from those reported by Mikkelsen and colleagues [5]. These were converted to mm9 build using Galaxy lift over tools and overlapped with peaks of Zfp57 enrichment. Average histone profiles around Zfp57 peaks were constructed using the cumulative probe trend function in SeqMonk.

*Zfp57-CGI methylation analysis*

In order to determine methylation status of ~23,000 CGIs in the mouse genome [6], we used published ES cell methylomes generated by RRBS reduced representation bisulphite sequencing analysis [7]. Coordinates of individual CpGs with read depth ≥5 were mapped to those of CGIs and mean methylation scores were then calculated across all CpGs mapped to the CGI. Only CGIs with ≥5 informative CpGs were analysed. Values for CGI methylation in GV oocyte, sperm and blastocyst were directly taken from the supplementary materials of [8]. Dot plot distributions of AMS values were plotted for groups of ZFP57 bound and randomly selected CGIs. Kruskal-Wallis test was used to compare these groups and Dunn’s post hoc test was used to make pairwise comparisons.

*Consensus motif search*

Genomic DNA sequence from the 158 ZFP57 peaks was extracted using Galaxy tools, repeat masked and supplied as input for MEME and NestedMICA motif inference algorithms. The following options were used: “motif width 6-18 bp”, “first order hidden-markov background model”, and “search in both strands”. Identification of individual motif occurrences were performed by searching the query sequences with regular expressions.

Scoring of the individual motif disruptions in the strain-specific binding sites was performed by aligning C57BL/6 and CastEiJ sequences. For each peak, the number of fully intact motifs were counted on each chromosome. A motifs was deemed to be disrupted if a SNP overlapped with one or more bases anywhere within the TGCCGCN consensus.

**Supplementary References:**

1. Langmead B, Trapnell C, Pop M, Salzberg SL: **Ultrafast and memory-efficient alignment of short DNA sequences to the human genome.** *Genome Biol* 2009, **10**:R25.

2. Keane TM, Goodstadt L, Danecek P, White M a., Wong K, Yalcin B, Heger A, Agam A, Slater G, Goodson M, Furlotte N a., Eskin E, Nellåker C, Whitley H, Cleak J, Janowitz D, Hernandez-Pliego P, Edwards A, Belgard TG, Oliver PL, McIntyre RE, Bhomra A, Nicod J, Gan X, Yuan W, van der Weyden L, Steward C a., Bala S, Stalker J, Mott R, et al.: **Mouse genomic variation and its effect on phenotypes and gene regulation**. *Nature* 2011, **477**:289–294.

3. Zhang Y, Liu T, Meyer CA, Eeckhoute J, Johnson DS, Bernstein BE, Nusbaum C, Myers RM, Brown M, Li W, Liu XS: **Model-based analysis of ChIP-Seq (MACS).** *Genome Biol* 2008, **9**:R137.

4. Quinlan AR, Hall IM: **BEDTools: a flexible suite of utilities for comparing genomic features.** *Bioinformatics* 2010, **26**:841–2.

5. Mikkelsen TS, Ku M, Jaffe DB, Issac B, Lieberman E, Giannoukos G, Alvarez P, Brockman W, Kim T-K, Koche RP, Lee W, Mendenhall E, O’Donovan A, Presser A, Russ C, Xie X, Meissner A, Wernig M, Jaenisch R, Nusbaum C, Lander ES, Bernstein BE: **Genome-wide maps of chromatin state in pluripotent and lineage-committed cells.** *Nature* 2007, **448**:553–60.

6. Illingworth RS, Gruenewald-Schneider U, Webb S, Kerr ARW, James KD, Turner DJ, Smith C, Harrison DJ, Andrews R, Bird AP: **Orphan CpG islands identify numerous conserved promoters in the mammalian genome.** *PLoS Genet* 2010, **6**.

7. Meissner A, Mikkelsen TS, Gu H, Wernig M, Hanna J, Sivachenko A, Zhang X, Bernstein BE, Nusbaum C, Jaffe DB, Gnirke A, Jaenisch R, Lander ES: **Genome-scale DNA methylation maps of pluripotent and differentiated cells.** *Nature* 2008, **454**:766–70.

8. Chotalia M, Smallwood S a, Ruf N, Dawson C, Lucifero D, Frontera M, James K, Dean W, Kelsey G: **Transcription is required for establishment of germline methylation marks at imprinted genes.** *Genes Dev* 2009, **23**:105–17.
